# Widespread Use of Non-productive Alternative Splice Sites in *Saccharomyces cerevisiae*

**DOI:** 10.1371/journal.pgen.1004249

**Published:** 2014-04-10

**Authors:** Tadashi Kawashima, Stephen Douglass, Jason Gabunilas, Matteo Pellegrini, Guillaume F. Chanfreau

**Affiliations:** 1 Department of Chemistry and Biochemistry and the Molecular Biology Institute, UCLA, Los Angeles, California, United States of America; 2 Bioinformatics Interdepartmental Program, UCLA, Los Angeles, California, United States of America; 3 Department of Molecular, Cellular and Developmental Biology, UCLA, Los Angeles, California, United States of America; University of California San Francisco, United States of America

## Abstract

*Saccharomyces cerevisiae* has been used as a model system to investigate the mechanisms of pre-mRNA splicing but only a few examples of alternative splice site usage have been described in this organism. Using RNA-Seq analysis of nonsense-mediated mRNA decay (NMD) mutant strains, we show that many *S. cerevisiae* intron-containing genes exhibit usage of alternative splice sites, but many transcripts generated by splicing at these sites are non-functional because they introduce premature termination codons, leading to degradation by NMD. Analysis of splicing mutants combined with NMD inactivation revealed the role of specific splicing factors in governing the use of these alternative splice sites and identified novel functions for Prp17p in enhancing the use of branchpoint-proximal upstream 3′ splice sites and for Prp18p in suppressing the usage of a non-canonical AUG 3′-splice site in *GCR1*. The use of non-productive alternative splice sites can be increased in stress conditions in a promoter-dependent manner, contributing to the down-regulation of genes during stress. These results show that alternative splicing is frequent in *S. cerevisiae* but masked by RNA degradation and that the use of alternative splice sites in this organism is mostly aimed at controlling transcript levels rather than increasing proteome diversity.

## Author Summary

Accurate gene expression requires the transfer of gene information from DNA to RNA. When DNA is transcribed into RNA, part of the RNA needs to be removed (spliced) to generate a proper copy of the genetic information. This process needs to be very accurate to preserve the genetic information that will be transferred into proteins. Our study shows that in baker's yeast, the splicing process does not always produce the correctly spliced products, as RNA splicing events frequently utilize incorrect splice sites. However, these deficient RNA molecules are eliminated from cells by a quality control mechanism to preserve the integrity of the genetic information. However, incorrect splicing is not useless, as it can be used to regulate the quantity of RNA that is generated.

## Introduction

Nonsense-mediated mRNA decay (NMD) is an RNA degradation system that degrades RNAs containing premature termination codons [1], [2]. In mammalian cells and higher eukaryotes, NMD can be used to regulate gene expression, for instance by reducing the level of alternatively spliced isoforms containing premature termination codons [3], [4], [5], [6], [7], [8]. This interplay between alternative splicing and NMD is involved in the autoregulation of SR proteins [3], [4], [5]. In addition to its function in regulating non-productively spliced isoforms, NMD is also used in a variety of eukaryotes to degrade unspliced pre-mRNAs that have escaped the splicing machinery [9], [10], [11], [12], [13]. Thus, NMD is widely involved in the proofreading of splicing efficiency and accuracy.

The yeast *Saccharomyces cerevisiae* has long been used as a model system to investigate the mechanisms of pre-mRNA splicing, as many components of the splicing machinery were identified through genetic screens in *S. cerevisiae* [14], and most splicing factors are highly conserved from yeast to mammalian cells [15]. Despite the presence of *c.a.* 330 intron-containing genes in *S. cerevisiae*, the prevalence of alternative splicing in this organism remains largely unexplored, as only a few examples of alternative splice site selection have been documented. The *SRC1* gene encodes an integral transmembrane protein, for which the use of an alternative 5′-splice site changes the number of passes through the membrane and ultimately the location of the C-terminal end of Src1p [16], [17]. Alternative 3′-splice site selection has been shown to regulate expression of the *APE2* gene according to a temperature-dependent secondary structure in the transcript [18]. A few other alternative 3′-splice sites have been described, and the use of some of these sites produces transcripts that are degraded by NMD [19]. Recent work analyzing alternative splicing across fungal species has shown that *S. cerevisiae* has lost some of the alternative splicing events through gene duplication and sub-functionalization of the duplicated genes, which are otherwise produced by alternative splicing in other species [20].

Using RNA-Seq analysis of strains mutated for NMD factors, we identify here a large number of alternative splice sites in *S. cerevisiae*. However, we show that splicing at these sites is generally non-productive because it introduces premature termination codons (PTC), leading to degradation of the transcripts by NMD. Non-productive splicing can be increased during environmental stress to contribute to a global regulatory mechanism that down-regulates transcripts levels in response to environmental cues. These results show that non-productive splice sites are widely used in *S.cerevisiae*, but that transcripts spliced at these sites are eliminated by RNA quality control mechanisms. Thus, while alternative splicing is frequently utilized in higher eukaryotes to generate proteome diversity, it is mainly used in *S.cerevisiae* as a means to regulate transcript levels.

## Results

### RNA-Seq reveals the accumulation of a large number of non-productive splice variants in NMD mutants

**Figure 1:**
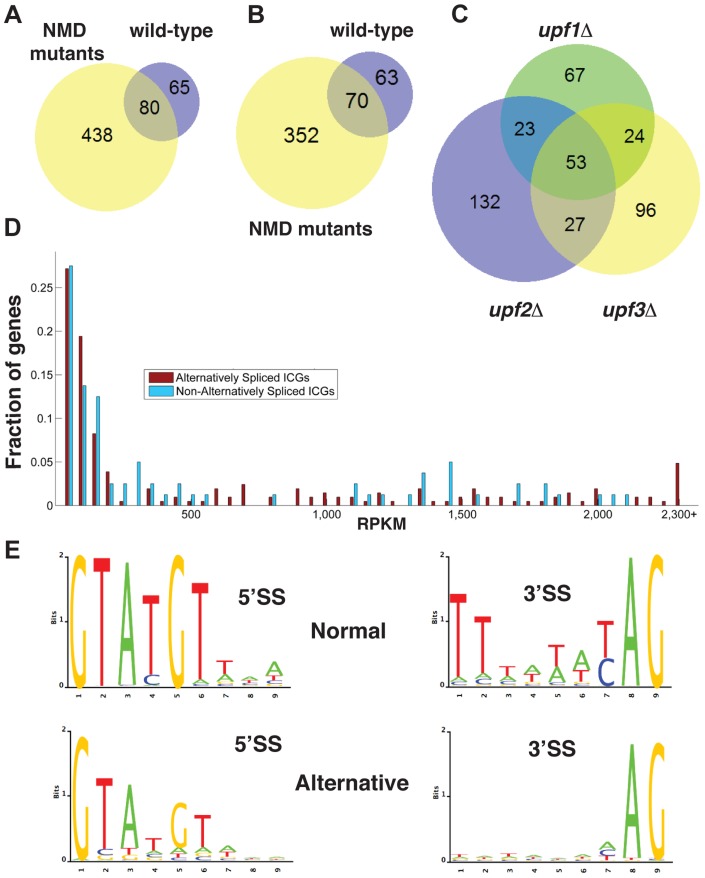
Bioinformatics analysis of alternative splice site usage in wild-type and NMD mutants. A. Venn diagram showing the overlap of alternative splice site usage between the wild-type and three NMD mutants pooled for all unique non-canonical splicing events (both PTC-generating and non-PTC-generating). B. Venn diagram showing the overlap of alternative splicing events between the wild-type and three NMD mutants pooled for all unique non-canonical splicing events resulting in a potential PTC. C. Venn diagram showing the overlap of alternative splicing events between the *upf1Δ*, *upf2Δ*, and *upf3Δ* strains for PTC-generating splicing events. D. Distributions of intron-containing gene transcripts showing alternative splicing events (red) or no alternative splicing events (blue) according to their overall abundance in RPKM. Transcripts for which the abundance was higher than 2,300 RPKM were grouped in the final bin. E. Sequence logo analysis of 5′- and 3′- splice sites for all normal and alternative splicing events detected by RNA-Seq in wild-type and NMD mutant strains.

We previously showed that NMD degrades unspliced transcripts arising from a large fraction of intron-containing genes in *S. cerevisiae*, due to suboptimal splice sites [12], [13], or upon splicing factor inactivation [21]. In addition, recent data showed that transcripts generated by the use of alternative 3′-splice sites can be degraded by NMD [19]. To gain further insights into the function of NMD in the proofreading of spliced isoforms, we performed RNA sequencing of mRNAs from wild-type and isogenic *upf1* ***Δ***, *upf2* ***Δ*** and *upf3* ***Δ*** strains defective for NMD. To identify transcripts spliced at alternative splice sites, we performed gapped alignment analysis of the RNA sequences (Table S1) using BLAT [22]. This analysis revealed numerous occurrences of spliced transcripts arising from previously unknown splice sites, in both WT and the NMD mutants. We will refer to these new splicing events as alternative splicing events, even if these are found in wild-type cells, and to the annotated splicing events as the normal or canonical splicing events. Alternative splicing events were detected more frequently in RNA samples obtained from the NMD mutants (Fig. 1A and 1B; Table S2), consistent with the fact that most of these alternative splicing events result in the introduction of a PTC, either by inducing a translational frameshift or by inserting an intronic PTC-containing sequence (Table S2). After adjusting for sequencing depth, *upf1* ***Δ***, *upf2* ***Δ*** and *upf3* ***Δ*** showed a 1.67, 1.72, and 1.90-fold enrichment in alternative splicing events and 1.59, 1.70, and 1.79-fold enrichment in PTC-generating alternative splicing events, respectively, versus wild-type (Table S2). NMD mutants showed an approximately 1.7-fold increase in unspliced mRNAs compared to the wild-type (Table S3) when considering reads that map to intronic and exon-intron regions, confirming our previous results from tiling arrays showing the involvement of NMD in eliminating unspliced transcripts genome-wide [12]. This enrichment for unspliced RNAs in NMD mutants is probably underestimated. Although there were 4-fold more reads that mapped only to intronic regions in NMD mutants compared to wild-type (Table S3), we observed an unanticipated high number of reads that mapped to exon-intron junctions in the wild-type strain (Table S3), which lowered the overall enrichment for unspliced RNAs in NMD mutants.

There was limited overlap in the alternative splicing events identified in the three *UPF* mutants (Fig. 1C), suggesting that the depth of our sequencing analysis was not sufficient to saturate identification of all alternative splicing events, particularly those occurring at low frequencies. The list of intron containing genes (ICG) for which we did not find the use of alternative splice sites is provided in Table S4, and the list of genes for which alternative splicing events were detected is shown in Table S5. 97 out of 304 intron containing genes analyzed did not exhibit alternative splicing (Table S4). Whether this reflects the absence of competing alternative sites or the lack of depth of our sequencing analysis remains to be determined.

To investigate if alternative splicing events are due to rare events or to splicing errors that occur randomly during transcript expression, we examined the abundance of ICG mRNAs that exhibited alternative splicing events and that of ICG mRNAs for which no alternative splicing events were detected (Fig. 1D). This analysis showed that some low abundance transcripts exhibited alternative splicing, while some high abundance transcripts did not (Fig. 1D). In addition, the median abundance of genes that showed alternative splicing was 117 RPKM, while the median abundance for genes with no alternative splicing events detected was 136 RPKM. Thus, even if the most highly expressed ICG (>2200 RPKM) all exhibited alternative splicing (Fig. 1D), genes with no alternative splicing were in general expressed at higher level than genes for which alternative splicing events were detected, showing no clear correlation between transcript abundance and the detection of alternative splicing events. We conclude that the detection of alternative splicing events in our RNA-Seq analysis is not an indirect consequence of the higher number of reads for highly-expressed transcripts.

The consensus sequences derived from the alternative splicing events identified in wild-type and all three mutants exhibited differences from the consensus sequences derived from the canonical (normal) splicing events (Fig. 1E). Alternative 5′-splice sites showed a relaxation of the conserved sequences, especially at positions 4 and 6 compared to the consensus obtained from the canonical splicing events. The 3′-splice sites used in alternative splicing events also showed a decrease in conservation of the polypyrimidine sequence preceding the conserved YAG, as well as a weaker conservation of the pyrimidine preceding the conserved AG dinucleotide (Fig. 1E). Thus, alternative splice sites identified by RNA sequencing showed a relaxed conservation, suggesting that these might correspond to lower efficiency splice sites, and possibly to regulated splicing events. Finally, we identified a number of alternative splicing events in either wild-type or NMD mutants that do not introduce a PTC and would potentially result in the production of proteins that differ from the SGD annotations. The list of these potential alternative proteins is presented in Table S6. However, we did not investigate these alternative protein forms further because most of the RNAs that would result in the production of these proteins were found in low abundance compared to those resulting in the production of the annotated proteins.

### Strategy for validation of alternative splicing events

**Figure 2:**
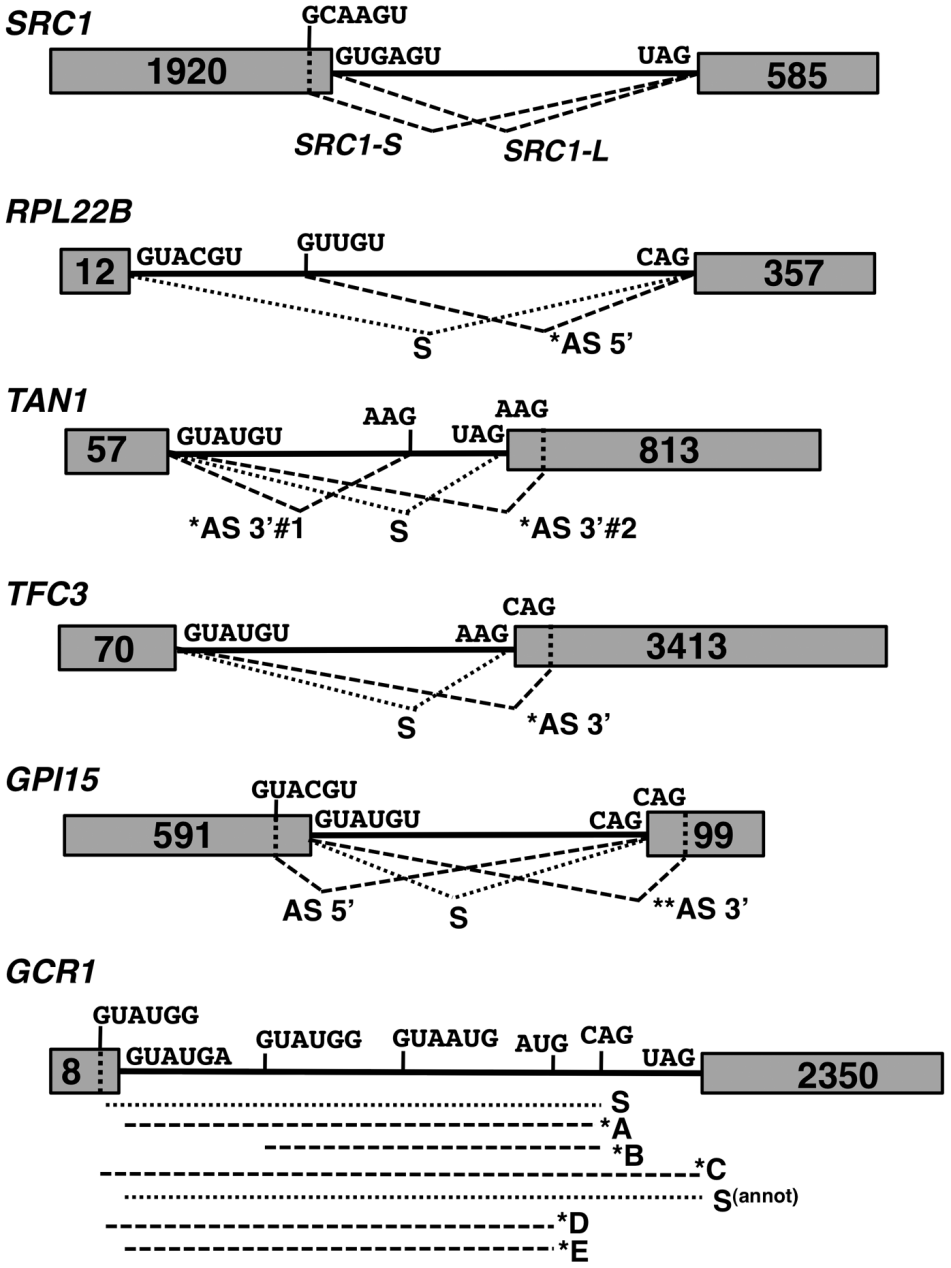
Spliced species produced from the *SRC1*, *RPL22B*, *TAN1*, *TFC3*, *GPI15* and *GCR1* genes. Species labeled with an asterisk are subject to NMD. Species labeled with two asterisks are predicted to be subject to NMD but were not observed to do so in subsequent experiments. The alternative 3′-SS of *SRC1* is located 4 nt upstream from the annotated 3′-SS. The alternative 3′-SS of *RPL22B* is located 64 nt downstream from the annotated 3′-SS. The alternative 3′-SS of *TAN1* are located 6 nt upstream and 7 nt downstream from the annotated 3′-SS. The alternative 3′-SS of *TFC3* is located 17 nt downstream from the annotated 3′-SS. The alternative 5′ and 3′-SS of *GPI15* are located 36 nt downstream and 14 nt upstream, respectively, from the annotated 5′ and 3′-SS. The alternative 3′-SS of *GCR1* are located 5 nt upstream (GUAUGG); 51 nt downstream (GUAUGG) and 627 nt downstream from the annotated 5′SS. The alternative 3′-SS of *GCR1* are located 40 nt upstream (AUG) and 17 nt downstream (CAG) from the annotated 3′-SS.

The previous RNA-Seq analysis revealed the potential widespread usage of alternative splice sites (SS). Figure 2 depicts specific mRNAs that were chosen for validation and further characterization. These transcripts were classified into three classes: those with 1) alternative 5′-SS; 2) alternative 3′-SS; and 3) a combination of both. Transcripts from class 1 included *RPL22B* as well as the previously reported *SRC1* [16]. Class 2 transcripts included genes encoding the RNA Polymerase III transcription factor *TFC3* with a downstream alternative 3′-SS, and the adenosine deaminase *TAN1* with two alternative 3′-SS flanking the normal 3′-SS. For the third class, we examined genes encoding the glycosylphosphatidylinositol biosynthetic enzyme *GPI15* and the transcriptional regulator *GCR1*. *GPI15* exhibited the use of an alternative 5′-SS with the normal 3′-SS, as well as the normal 5′-SS with an alternative 3′-SS (Fig. 2). *GCR1* showed a more complex splicing pattern with multiple combinations of 5′ and 3′-SS (Fig. 2).

**Figure 3:**
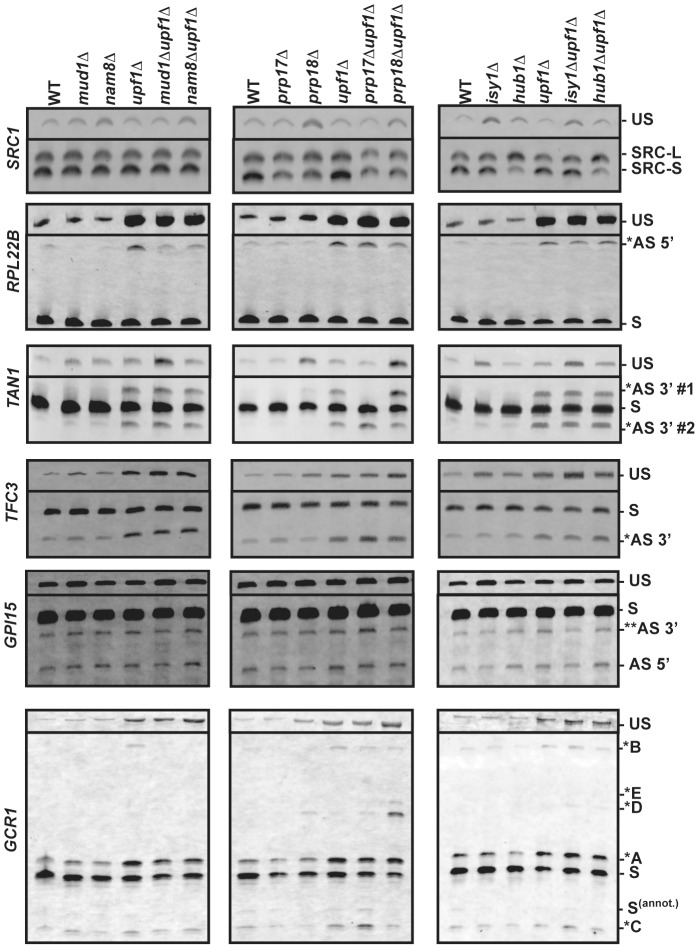
RT-PCR analysis of alternatively spliced products for *SRC1*, *RPL22B*, *TAN1*, *TFC3*, *GPI15* and *GCR1* in wild-type, NMD and various splicing mutants. The unspliced (US) species is also shown on top. The middle portions of the gel where no species were visible have been removed. In all cases, RT-PCR was performed with a Cy3-labeled primer. The labeling of the different alternatively spliced forms is according to the nomenclature shown in Figure 2.

We analyzed alternative splicing events by RT-PCR using Cy3-end labeled primers, which allowed for relative comparison of the abundance of spliced and unspliced species, regardless of their size. Because we lacked an adequate size marker for Cy3 detection, the same RT-PCR analyses were initially performed with ^32^P-end labeling with an appropriate ^32^P-labelled ladder (data not shown) to confirm the sizes of all RT-PCR products and correlate the data back to gels obtained with Cy3-labeled primers. In addition to the wild-type and NMD-deficient *upf1* ***Δ*** strains, we analyzed the phenotypes of a number of *S. cerevisiae* splicing mutants. Knockout mutants of genes encoding Mud1p and Nam8p were chosen for their association with the U1 snRNP and role in 5′-SS selection [23], [24], [25], [26]. The *HUB1* knockout was also included, as Hub1p was recently implicated in 5′-SS selection for *SRC1* [17]. Prp17p and Prp18p were selected for their involvement in the second step of splicing and potential effects on 3′-SS selection [27], [28]. Finally, Isy1p was also included as a potential splicing fidelity factor [29]. The splicing profiles were analyzed for each of the genes mentioned above in each of these mutant strains by fractionation of the RT-PCR products on polyacrylamide gels (Fig. 3). For the splicing mutants for which the splicing pattern differed from the wild-type, additional RT-PCR experiments were performed in triplicate from three independent cultures and quantitated, as shown in Supporting Figures S1, S2, S3, S4 and S6.

### RT-PCR analysis confirms the involvement of Prp17p and Hub1p in *SRC1* alternative splicing

As a first step in validating our RT-PCR strategy, we focused on *SRC1*, which exhibits two possible 5′-SS (Fig. 2) and for which previous studies have demonstrated the roles of various splicing factors [16], [17], [30]. RT-PCR analysis of *SRC1* splice variants confirmed the use of these two alternative 5′-SS (Fig. 3). Wild-type samples showed a 60/40 ratio of *SRC1-S*/*SRC1-L* (Fig. S1), consistent with previous reports [16], [17], [30]. Samples from the *upf1* ***Δ*** mutant showed a pattern similar to wild-type (Fig. 3; Fig. S1), indicating that both variants are stable and not targeted by NMD. This result is consistent with our RNA-Seq analysis, which showed high sequence counts for both forms in all strains. Samples from the *nam8* ***Δ*** strain showed a slight increase in the level of unspliced transcripts (Fig. 3) due to reduced splicing efficiency [31]. The *prp17* ***Δ*** and *prp18* ***Δ*** mutants both showed a slight increase in the usage of *SRC1*-L 5′-splice site relative to the *SRC-S* 5′-splice site (1.4 and 1.3 fold, respectively), as suggested previously for the *prp17* ***Δ*** mutant at the protein level [17], and the *prp18* ***Δ*** mutant also exhibited an increase in unspliced precursors accumulation, consistent with previous results for other transcripts [21]. The *isy1* ***Δ*** mutant strain exhibited a clear accumulation of unspliced pre-mRNAs (Fig. 3), in agreement with the documented role of Isy1p in maintaining the proper conformation needed for the 1^st^ step of splicing [29]. Hub1p inactivation resulted in a 3-fold reduction in the amount of *SRC1-S*, coinciding with an increase in *SRC1-L* (Fig. S1, Fig. 3), consistent with previous reports [17], [30]. This reduction was also observed in the context of the *upf1* ***Δ*** mutant (Fig. S1, Fig. 3). Thus, the results described above confirmed the previously described effects of various splicing mutants on *SRC1* splicing patterns and showed that our RT-PCR strategy is effective in analyzing the impact of specific splicing factors on splice site usage..

### Efficient use of the non-productive 5′-splice site of *RPL22B* is strongly dependent on the U1 snRNP components Nam8p and Mud1p

*RPL22B* showed the presence of an alternative 5′-SS in the intronic sequence, which unlike *SRC1*, yields a PTC-containing transcript potentially targeted to NMD (Fig. 2). This alternatively spliced transcript was almost 10-fold more abundant in the *upf1* ***Δ*** mutant (Fig. 3; Fig. S2), further suggesting that it is targeted by NMD. We also detected a large accumulation of unspliced species in the *upf1* ***Δ*** mutant, indicating inefficient recognition of this splicing substrate. This may be the result of both the normal (GUACGU) and alternative (GUUUGU) 5′-SS having non-consensus sequences (see below). Interestingly, the abundance of the alternatively spliced product was found to decrease by two to three folds when Nam8p or Mud1p were inactivated in the context of the *upf1* ***Δ*** deletion (Fig. 3; Fig. S2). The deletion of either one of these two factors might hinder the ability of the U1 snRNP to bind to the alternative suboptimal 5′ GUUUGU splice site of *RPL22B*, resulting in decreased usage. This is consistent with the known roles of Mud1p and Nam8p in the first step of splicing [25], and suggest their direct involvement in modulating 5′-SS selection of *RPL22B*. By contrast, no major changes were observed in the *prp17* ***Δ***, *prp18* ***Δ***, *isy1* ***Δ***, *hub1* ***Δ*** mutants, either alone or in combination with the *upf1* ***Δ*** deletion (Fig. 3), showing the specificity of the effects detected with Nam8p and Mud1p. Thus, *RPL22B* exhibits two competing suboptimal 5′-SS, one of which is highly sensitive to perturbations in the U1 snRNP. The functional significance of the alternative 5′-SS of *RPL22B* in regulating transcript levels is investigated further below.

### A novel role for Prp17p in promoting the use of branchpoint proximal alternative 3′-splice sites

Gapped sequence alignment showed that *TFC3* exhibits an alternative CAG 3′-SS 17 nt downstream of the annotated AAG (Fig. 2). This product can be detected in samples from the wild-type and splicing mutants, but is 4.5-fold more abundant in the context of the *upf1* ***Δ*** deletion, showing that a large fraction of this product is degraded by NMD (Fig. 3; Fig. S3). This non-productive isoform amounts to 27% of all spliced products (Fig. S3), showing that a significant fraction of splicing generates NMD-targeted, non-productive transcripts. We observed a slight accumulation (1.7 fold) of the downstream alternative 3′-splice product in the *prp17* ***Δ*** mutant (Fig. 3; Fig. S3), showing that this second step splicing factor contributes to reducing the use of this alternative 3′-SS. As expected, inactivation of the first step splicing factors Mud1p or Nam8p had no effect on the pattern of 3′-SS selected (Fig. 3).

*TAN1* exhibits a more complex alternative 3′-SS pattern, where a canonical UAG 3′-SS is flanked by two alternative 3′ AAG sequences (Fig. 2). The use of either of these sites would generate PTC-containing transcripts. The upstream AAG (AS 3′ #1) is only 6 nt away from the canonical 3′-SS. The retention of 6 nt of intronic sequence would maintain the proper reading frame but would result in a PTC because the UAG sequence of the normal 3′-splice site corresponds to an in-frame stop codon [32]. The downstream AAG (AS 3′ #2) is 7 nt downstream of the normal 3′-SS, resulting in a frameshift-induced PTC. RT-PCR analysis of the wild-type and *upf1* ***Δ*** strains confirmed the RNA-Seq data by showing that these two alternative splice products are detected at extremely low levels, unless NMD is inhibited (Fig. 3; Fig. S4). In samples from the *upf1* ***Δ*** strain, the two alternatively spliced products accumulate to similar amounts, and both species are detected at lower levels than the normal spliced product (20% of all spliced products; Fig. S4), possibly because these two suboptimal AAG sites do not compete well with the consensus canonical UAG site. Strikingly, the usage of these alternative 3′ splice sites was dramatically altered when Prp17p or Prp18p were inactivated. Inactivating Prp17p resulted in an increase in the use of the downstream alternative 3′-SS (AS 3′#2), while the upstream alternative 3′-SS (AS 3′#1) was no longer used (Fig. 3; Fig. S4), showing a role of Prp17p in enhancing the use of upstream, branchpoint proximal 3′-SS. By contrast, Prp18p inactivation resulted in increased usage of the alternative 3′-SS most proximal to the branch point sequence (AS 3′ #1; Fig. 3; Fig. S4). This product is barely detectable in the wild-type strain but can be observed in the *prp18* ***Δ*** strain (Fig. 3), and inactivation of Prp18p in the context of the *upf1* ***Δ*** deletion resulted in a 3-fold increase in the abundance of this species (Fig. S4). The effect of Prp18p on this 3′-SS might be due to the identity of the sequences immediately following the 3′-SS, which have been shown to influence 3′-SS selection in the absence of a functional Prp18p [33]. Isy1p inactivation resulted in an increase of unspliced species in a similar fashion to *SRC1* discussed above; however there was no effect of Isy1p, Hub1p, Mud1p and Nam8p on alternative 3′-SS selection of *TAN1* (Fig. 3), showing the specificity of the effects observed with Prp17p and Prp18p. Finally, unspliced *TAN1* transcripts were generally not affected by NMD, except in the context of a *mud1* ***Δ*** mutant strain (Fig. 3). This observation is consistent with a recent report showing that *TAN1* unspliced transcripts are retained in the nucleus by the RES complex, and are subject to NMD only when the RES complex is inactivated [34]. Overall, analysis of *TFC3* and *TAN1* alternative 3′-SS patterns show that Prp17p and Prp18p have antagonistic roles in the selection of upstream and downstream 3′-SS *of TAN1*, and highlight the importance of Prp17p in enhancing the use of 3′-SS located closer to the branchpoint.

### Alternative splicing patterns of *GPI15* and *GCR1* reveal the production of alternative non-functional protein products and the use of a non-canonical AUG 3′-splice site repressed by Prp18p

*GPI15* in an interesting case where the two alternatively spliced products identified by our RNA-Seq analysis are not targeted by NMD. The use of an alternative GUACGU 5′-splice site results in the deletion of 30 nucleotides from the 3′ end of exon 1 (Fig. 2), which maintains the open-reading frame but generates a truncated protein. However, the protein product resulting from translation of this alternatively spliced product is likely to be non-functional, as this truncation removes a stretch of 10 amino acids at positions 187–197 in the most highly conserved region of this protein [35]. This transcript can be detected in samples from the wild-type and the splicing factor mutants, and does not vary in intensity in the context of *upf1* ***Δ***, indicating that it is not targeted by NMD (AS 5′, Fig. 3). In contrast, the alternatively spliced transcript generated by use of a downstream CAG 3′-SS results in a PTC. However, this PTC-containing transcript would exhibit a short 85 nt 3′-UTR, which might render it insensitive to NMD as suggested by the *faux 3′ UTR* model [36], [37]. Indeed, the abundance of this transcript was not increased in the *upf1* ***Δ*** mutant (Fig. 3). In addition, this transcript is expected to yield a non-functional protein due to C-terminal truncation and deletion of amino acids within the most conserved region of the protein [35]. Analysis of the pattern of selection of these two alternatively spliced transcripts in the various splicing mutants did not reveal any major effect of these mutants (Fig. 3) in contrast to the effects described above for *RPL22B*, *TAN1* or *TFC3*. However, there was a slight increase in the use of the downstream alternative 3′-SS in the *prp17* ***Δ*** *upf1* ***Δ*** strain, consistent with the role of Prp17p in favoring the upstream 3′-SS, as described above for *TAN1* and *TFC3*.

*GCR1* showed the most complex splicing pattern of all transcripts analyzed. Gapped alignments identified an intronic GUAUGG alternative 5′-SS as well as an upstream CAG alternative 3′-SS (Fig. 2). In addition to these alternative splice sites identified by RNA-Seq, RT-PCR revealed the use of an additional GUAUGG alternative 5′-SS staggered 5-nt upstream of the normal 5′-SS and of a non-canonical AUG alternative 3′-SS 23 nt upstream of the other alternative 3′-SS (Fig. 2). The use of all of these sites was confirmed by RT-PCR, cloning and Sanger sequencing (see below and Fig. S5). The fact that some alternative splice sites escaped identification by mRNA sequencing indicates that a greater depth of coverage has the potential to identify even more alternative splice sites.

Based on *GCR1* annotation, the canonical spliced mRNA would use the GUAUGA 5′-SS along with the most downstream UAG 3′-SS (Fig. 2). This product (labeled as S^(annot.)^ in Fig. 2 and 3), however was detected at very low levels (Fig. 3). The major spliced product observed resulted from the use of the most upstream GUAUGG 5′-SS and of an upstream CAG 3′-SS (labeled “S” in Fig. 2 and 3). This splicing event does not introduce a PTC and results in a protein that is very similar to the translation product of the annotated spliced transcript S^(annot.)^. The annotated amino acid sequence of *GCR1* from position 2 to 4 is VCT. In the major spliced product S, this sequence is replaced by QTSVDST. Thus, most of the protein is identical, except for a few N-terminal amino acids which are not expected to affect Gcr1p function, as all *GCR1* mutations with phenotypic effects have been mapped to a region downstream of this short sequence stretch [38], [39], [40]. Based on the relative abundances of S and S^(annot.)^, it is clear that S, and not S^(annot.)^ is the main spliced product for the *GCR1* gene.

In addition to this major spliced product, we also detected a series of alternatively spliced products degraded by NMD (as denoted by asterisks in Fig. 2 and 3). Splicing from the annotated GUAUGA 5′ splice site combined with the upstream CAG 3′ splice site resulted in a PTC-containing transcript labeled as *A in Figs. 2 & 3. This transcript is degraded by NMD, as higher amounts are observed in all the strains containing a *upf1* ***Δ*** deletion, and it is the most abundant of all *GCR1* alternatively spliced products subject to NMD (Fig. 3; Fig. S6). Another product is generated from combining the upstream GUAUGG 5′-SS with the most downstream UAG 3′-SS (*C in Fig. 2). This splicing event results in a PTC, as it introduces a translational frameshift, which is not detected until the 43rd amino acid is translated. The corresponding transcript accumulates at low abundance in all samples and appears to be targeted by NMD, as its abundance increases slightly in all *upf1* ***Δ*** strains. In addition, the use of this most downstream 3′-SS increases almost 4-fold in the *prp17* ***Δ*** *upf1* ***Δ*** strain when compared to the *upf1* ***Δ*** control (Fig. 3; Fig. S6). Because the 3′-SS used to generate this transcript corresponds to the most downstream one, this observation provides another example of the importance of Prp17p in favoring the selection of upstream 3′-SS, as shown above for *TFC3*, *TAN1* and to a lesser extent *GPI15*.

Another PTC-containing transcript that is degraded by NMD results from splicing of the downstream intronic GUAUGG 5′-SS with the CAG 3′-SS, (labeled *B in Fig. 2 and 3). This product is faint, but detectable in all cases of NMD deactivation, except in combination with *nam8* ***Δ*** or *mud1* ***Δ***, most likely because this 5′-SS has a higher sensitivity to U1 snRNP perturbations, as described above for *RPL22B*. Analysis of other mutants did not reveal any major influence on the pattern of 5′- or 3′-SS selection. Like *SRC1*, *GCR1* exhibits two staggered 5′ splice sites. However, unlike for *SRC1*, Hub1p has no influence on their selection (Fig. 3).

A final set of NMD targets are produced by the use of the two most upstream 5′-SS with a highly unusual alternative AUG 3′-SS in the intronic sequence (labeled *D and *E in Figs. 2 &3). Interestingly, these products were only detected in the absence of Prp18p, suggesting that this factor is essential in preventing the use of this non-canonical 3′-SS. The use of this highly unusual AUG 3′ splice site was unambiguously confirmed through sequencing and RT-PCR analysis of RNAs derived from *prp18* ***Δ*** *upf1* ***Δ*** samples (Fig. S5). The ATPase Prp22p has been implicated in the fidelity of 3′-SS selection [41]. Because Prp18p functions upstream from Prp22p during the late stages of splicing [42], it is possible that the absence of Prp18p might indirectly hinder the function of Prp22p in proofreading 3′-SS selection, and that the use of this unusual 3′-SS might be the consequence of a reduced Prp22p function in the absence of Prp18p. To test this hypothesis, we analyzed *GCR1* splicing in a *prp22-1* mutant. RT-PCR analysis showed that the spliced product generated from the use of the AUG 3′-SS did not accumulate in a *prp22-1* splicing mutant (Fig. S7). Thus, the accumulation of species resulting from the use of this unusual 3′-SS in the *prp18* ***Δ*** *upf1* ***Δ*** samples is not an indirect consequence of hindered Prp22p function. The discovery of the splicing at this unusual 3′-SS sequence reveals the importance of Prp18p in ensuring proper 3′-SS selection for *GCR1* and in repressing the use of non-canonical 3′-SS sequences.

### Alternatively spliced species of *RPL22B* and *GCR1* increase during stress conditions

**Figure 4:**
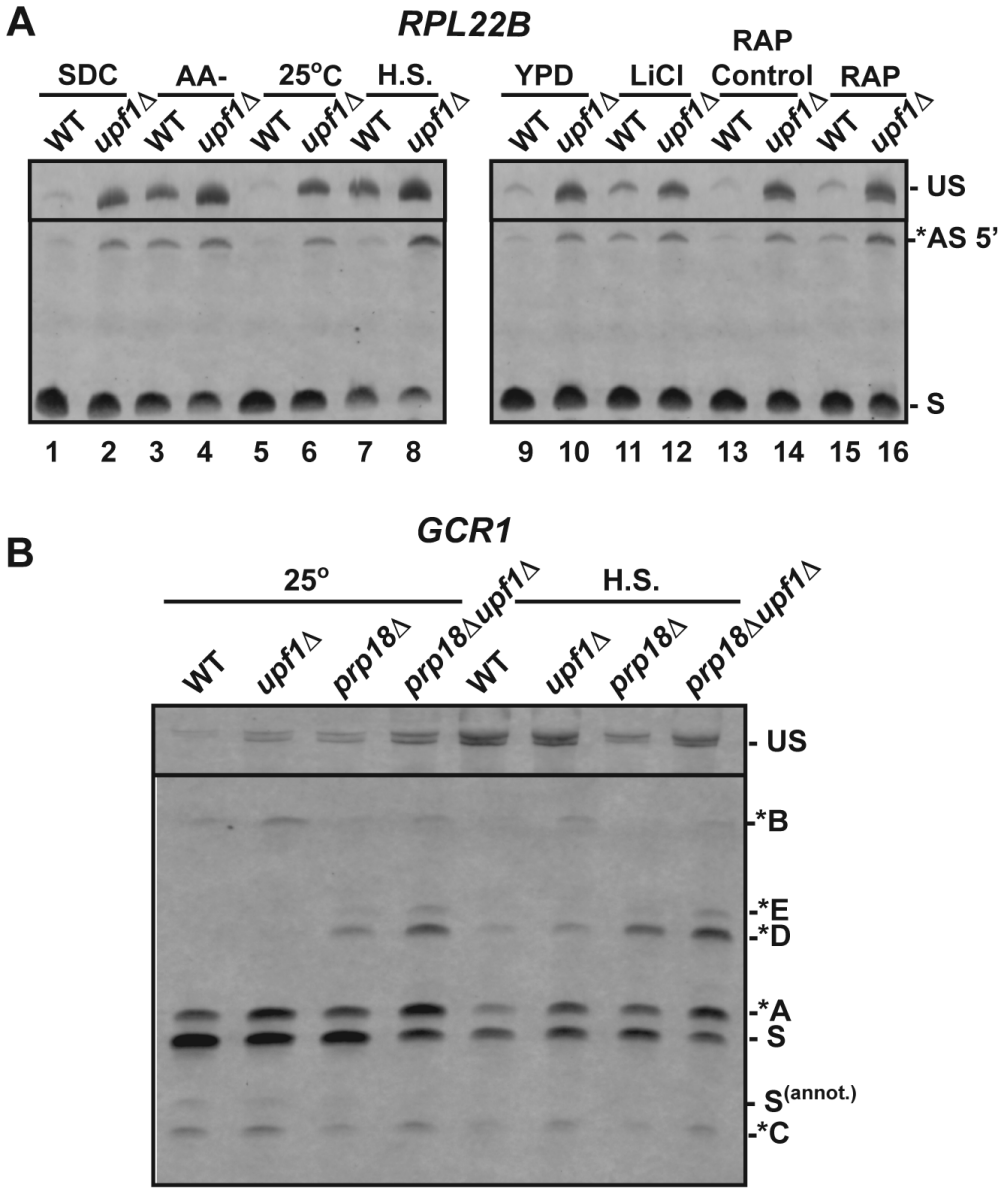
RT-PCR analysis of alternatively spliced products under stress conditions. A. Analysis of *RPL22B* in various stress conditions. Shown are the RT-PCR products obtained from the wild-type or *upf1Δ* mutant strain after growth in the following conditions: SDC, synthetic define complete medium at 30°C; -AA, 10 minutes in SDC medium at 30°C lacking amino acid (-AA); 25°C, log phase at 25°C in YPD; H.S, 20 minutes at 42°C in YPD; YPD: log phase at 30°C in YPD; LiCl, incubation with 300 mM LiCl in YPD at 30°C for 10 minutes; RAP control, see Materials and Methods; RAP, treatment with Rapamycin for 20 minutes. B. RT-PCR analysis of *GCR1* alternative splicing in heat-shock conditions. Labeling of the different species is similar to that of Figures 2 and 3.

The previous results validated our prediction that transcripts generated from the use of alternative non-productive splice sites are degraded by NMD and revealed the role of specific splicing factors in governing the choice between alternative sites. Strikingly, the sequence of some of these non-productive splice sites was found to be conserved across closely related yeast species (Fig. S8, *RPL22B* and Fig. S9, *TAN1*). Because the level of sequence conservation in intronic sequences is usually very low (Fig. S8, S9), these peaks in sequence conservations for intronic alternative splice sites might reflect their functional importance. We hypothesized that the use of some of these alternative splice sites which lead to degradation by NMD might be favored under certain conditions to down-regulate gene expression. To test this hypothesis, we monitored changes in the splicing patterns of *RPL22B*, *TAN1*, and *TFC3* under stress conditions such as amino acid starvation, heat shock, LiCl-mediated hyperosmotic stress, and rapamycin treatment, as these have been reported to elicit diverse responses in the expression of intron containing genes [43], [44]. In addition, various stresses cause down-regulation in ribosomal protein gene expression (many of which contain introns), presumably to relieve the cell of massive energy requirements of ribosome biogenesis and focus those resources into regulations that are the most appropriate in response to the current stress condition [45], [46], [47]. After 10 minutes of amino acid depletion, *RPL22B* showed an increase in unspliced species as well as well as a 4.5-fold increase in the level of the alternatively spliced product when compared to the SDC or YPD media controls (Fig. 4A; Fig. S10). In the *upf1* ***Δ*** strain shifted to amino acid starvation conditions, the levels of the alternatively spliced product increased compared to the wild-type strain grown in the same conditions, as would be expected when NMD transcripts are no longer degraded (Fig. 4A lanes 2 and 4). The fact that the level of the alternatively spliced transcript is 2.5-fold higher in the *upf1* ***Δ*** sample than in the wild-type sample under amino acid starvation conditions (Fig. S10) argues that the increase in the abundance of these species in the wild-type strain in these conditions is not due to NMD inhibition in these conditions, but that a change in splice site selection occurs that favors the use of the alternative splice site. Significantly, amino acid starvation did not change the levels of the alternatively spliced species of *TAN1* and *TFC3* that are normally subject to NMD (Fig. S11). This observation provides further evidence that the increase in the amount of alternatively spliced *RPL22B* transcript observed during amino acid starvation is due to a switch in splice site selection and not to an inhibition in NMD, since the level of alternatively spliced species of *TAN1* and *TFC3* that are normally degraded by NMD is unaffected in the same conditions.

We next investigated the effect of a 20 minute heat shock at 42°C on splicing patterns. Under these conditions and in the wild-type strain, *RPL22B* showed an increase in unspliced as well as a decrease in the relative amount of the normal spliced product (Fig. 4A lane 5 vs. 7). More importantly, the NMD defective strain *upf1* ***Δ*** showed an even larger increase in unspliced pre-mRNAs, as well a large accumulation of the alternatively spliced product that coincides with a decreased amount of canonical spliced product (Fig. 4A lane 6 vs. 8). In these conditions, the alternatively spliced product now corresponds to more than half of all spliced species (Fig. S10). Under heat shock, this alternatively spliced product is 4-fold more abundant in the *upf1* ***Δ*** strain than in the wild-type strain. These higher levels upon NMD inactivation show that the increased accumulation of these species under heat shock is not due to a decrease in NMD efficiency. Rather, this result shows that the use of the alternative splice site is being favored in heat shock conditions. By contrast, *TFC3* and *TAN1* exhibited an accumulation of unspliced species, but decreased levels of both the canonical and alternatively spliced species (Fig. S11), consistent with a general inhibition of pre-mRNA splicing under heat shock [48], [49]. Thus the pattern of alternatively spliced species of *TFC3* and *TAN1* that are subject to NMD is very different from that of *RPL22B*, further proving that the accumulation of the alternatively spliced *RPL22B* transcript under heat shock conditions described above is not due to a general stabilization of spliced forms degraded by NMD.

Like heat shock, rapamycin treatment was shown to result in an inhibition of ribosomal proteins mRNA splicing based on microarray experiments [43]. Within 20 minutes of rapamycin treatment, *RPL22B* indeed showed trends similar to those observed in heat shock, but to a lesser degree, with an increase of unspliced species and of alternatively spliced *RPL22B* species (Fig. 4A), but no effect on the alternatively spliced *TAN1* and *TFC3* transcripts (Fig. S11). Hyperosmotic shock (300 mM LiCl exposure for 10 min) only resulted in minimal effects; there were no changes observed for *TFC3* and *TAN1* targets under these stress conditions (Fig. S11), and *RPL22B* showed only a slight increase in unspliced but the levels of spliced transcripts remained similar. Thus, *RPL22B* exhibits regulated use of its alternative 5′-splice site, mostly under amino acid starvation and heat shock conditions, while other transcripts such as *TFC3* and *TAN1* did not exhibit any change in their alternative splicing profiles.

Because *GCR1* exhibited a very complex splicing pattern, especially in the absence of Prp18p, and because heat shock conditions resulted in the most dramatic changes in splicing for *RPL22B*, we next investigated the effect of heat shock on *GCR1* splicing in the wild-type, *upf1* ***Δ***, *prp18* ***Δ*** and *prp18* ***Δ*** *upf1* ***Δ*** mutants (Fig. 4B). Under heat-shock, we detected a general inhibition of splicing, consistent with the data described above. However, we also observed an increase of the abundance of the A* form relative to the normal spliced product S, indicative of a switch from the normal GUAUGG site to the GUAUGA site. The absence of Prp18p resulted in an increase of the use of the non-canonical AUG site (*D species) in heat shock conditions, and this product now constituted one third of all spliced species. Thus we conclude that *GCR1*, like *RPL22B*, exhibits a switch in splice site selection during heat shock, and that Prp18p limits splicing at this non-canonical AUG site under stress conditions.

### The alternative, suboptimal 5′-splice site of *RPL22B* contributes to the global down-regulation of *RPL22B* in stress conditions

**Figure 5:**
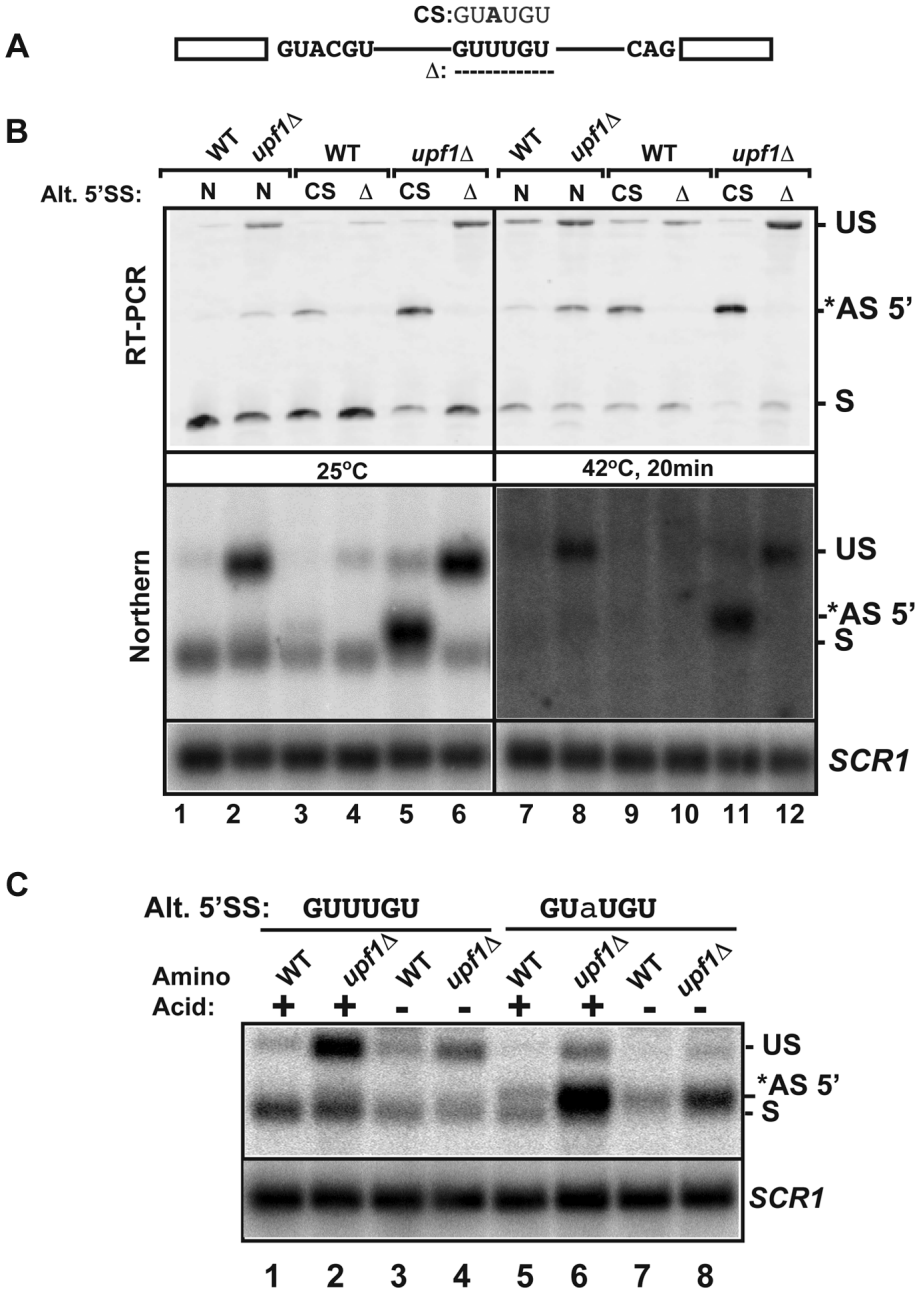
Effects of mutations of the *RPL22B* alternative 5′ splice site on *RPL22B* splicing and expression in normal and stress conditions. A. Organization of the *RPL22B* precursor, with the normal and alternative 5′-splice sites. Shown are the mutations to the consensus sequence (CS) GUAUGU or the deletion that entirely removes the GUUUGU sequence. B. Analysis of the effect of these mutations on *RPL22B* splicing and expression at normal temperatures (25°C) or after a 20 min heat shock at 42°C. N, natural 5′-splice site (GUUUGU); CS, consensus sequence (GUAUGU); Δ = deletion of the alternative 5′-splice site. Top panel: RT-PCR analysis. Bottom panel: northern blot analysis. US, *AS-5′, and S indicate the location of the products corresponding to the unspliced, alternatively spliced and normal spliced products, respectively. For the northern blot, *SCR1* was used as a loading control. C. Analysis of the effect of the *RPL22B* alternative splice site consensus mutation on *RPL22B* expression during amino acid starvation. Shown is a northern blot of RNA samples extracted from the indicated strains grown at 30°C in normal synthetic define complete (SDC) medium with amino acid (+) or in SDC medium lacking amino acid (−) for 10 minutes. Strains contained either the natural GUUUGU sequence at the alternative 5′-splice site of RPL22B, or the consensus GUaUGU sequence. The nucleotide mutated is highlighted in lower case. Labeling of the different species is similar to that of panel B. *SCR1* was used as a loading control.

To further analyze the importance of the alternative 5′-splice site of *RPL22B* on its splicing patterns and expression during normal and stress conditions, we investigated the effect of mutations of this alternative 5′-SS. The suboptimal GUUUGU alternative 5′-splice site was either deleted or mutated to the consensus GUAUGU sequence at the endogenous chromosomal locus (CS, consensus mutation and **Δ**, deletion, Fig. 5A). Changing the alternative 5′-SS to the consensus GUAUGU sequence resulted in detectable amounts of alternatively spliced products at 25°C, even in a functional NMD background (Fig. 5B), suggesting that the suboptimal GUUUGU sequence contributes to the low usage of this alternative site in normal conditions. Inactivation of Upf1p in this context showed that 70% of all spliced species were now being produced by splicing from the alternative consensus site (Fig. 5B; lane 5; Fig. S12 for quantitation), and that splicing efficiency was improved, as shown by a decrease in unspliced species. By contrast, deleting the alternative splice site resulted in higher amount of unspliced transcripts, especially in the *upf1* ***Δ*** background. Thus, deleting the alternative 5′-splice site of *RPL22B* is not sufficient to improve splicing at the normal splice site, possibly because of the suboptimal sequence of the normal *RPL22B* 5′-splice site. In addition to RT-PCR, the same strains were analyzed by northern blot (Fig. 5B, bottom panel), which yielded results similar to those obtained by RT-PCR. These results show that increasing the strength of the alternative 5′-SS of *RPL22B* is sufficient to enhance the overall splicing efficiency of this transcript, while deleting this site results in an overall increase of unspliced RNAs. Under heat shock and NMD inactivation, this effect was even more prominent, as mutation of the alternative splice site to the consensus resulted in the alternatively spliced product being the major spliced species (Fig. 5B, lane 11). Thus, under heat shock conditions, *RPL22B* transcripts bearing the consensus alternative splice site mutation are now spliced almost exclusively at this site. Analysis of the mutant with a deletion of the alternative 5′-SS under heat shock conditions showed that the use of the normal 5′-SS is not increased at elevated temperatures when the competing alternative 5′-SS has been eliminated (Fig. 5A and 5B lane 12). This mutant shows a larger accumulation of unspliced *RPL22B* transcript, hinting that the normal process of spliceosome assembly is perturbed on this transcript during heat shock, possibly due to the suboptimal 5′-SS. To obtain a more quantitative assessment of transcript levels, rather than just assessing the ratio between the different spliced forms, we analyzed the same samples by northern blot. This analysis showed that cells treated in heat shock conditions resulted in much weaker signal than in the samples obtained from cells grown at 25°C, consistent with a general down-regulation of ribosomal protein genes under stress [43], [44], [45], [46], [47]. Upon NMD inactivation, we observed a rescue of transcript levels, which mostly corresponded to unspliced RNAs and to some alternatively spliced transcripts (Fig. 5B). However, changing the alternative 5′-SS to a consensus sequence in the context of NMD inactivation was sufficient to recover a large amount of spliced transcripts (Fig. 5B, lane 11, lower panel). To investigate if this effect was specific to heat shock or is also observed during other stresses, we analyzed the expression of wild-type and mutated forms of *RPL22B* during amino acid starvation (Fig. 5C). The results observed during amino acid starvation were similar to those described during heat shock, with a large increase in the level of spliced transcripts upon changing the alternative 5′-splice site to the consensus sequence. We also observed an increase in the use of the alternative 5′-SS under amino acid starvation (Fig. S13). Interestingly, shifting the Upf1p-inactivated strain with the alternative 5′-splice site consensus sequence from SDC to amino acid starvation conditions resulted in only a minor increase of the use of the alternative 5′-SS, possibly because the level of transcripts spliced at that site is already very high in the *upf1* **Δ** strain in normal conditions (75%; Fig. S13). In conclusion, these results show that low splicing efficiency due to the suboptimal normal and alternative 5′ splice sites of *RPL22B*, combined with NMD degradation of the unspliced and alternatively spliced forms contribute to the general decrease in *RPL22B* levels as a means to rapidly halt production of this ribosomal protein under various stress conditions.

### Usage of the alternative 5′-splice site of *RPL22B* is influenced by promoter identity

**Figure 6:**
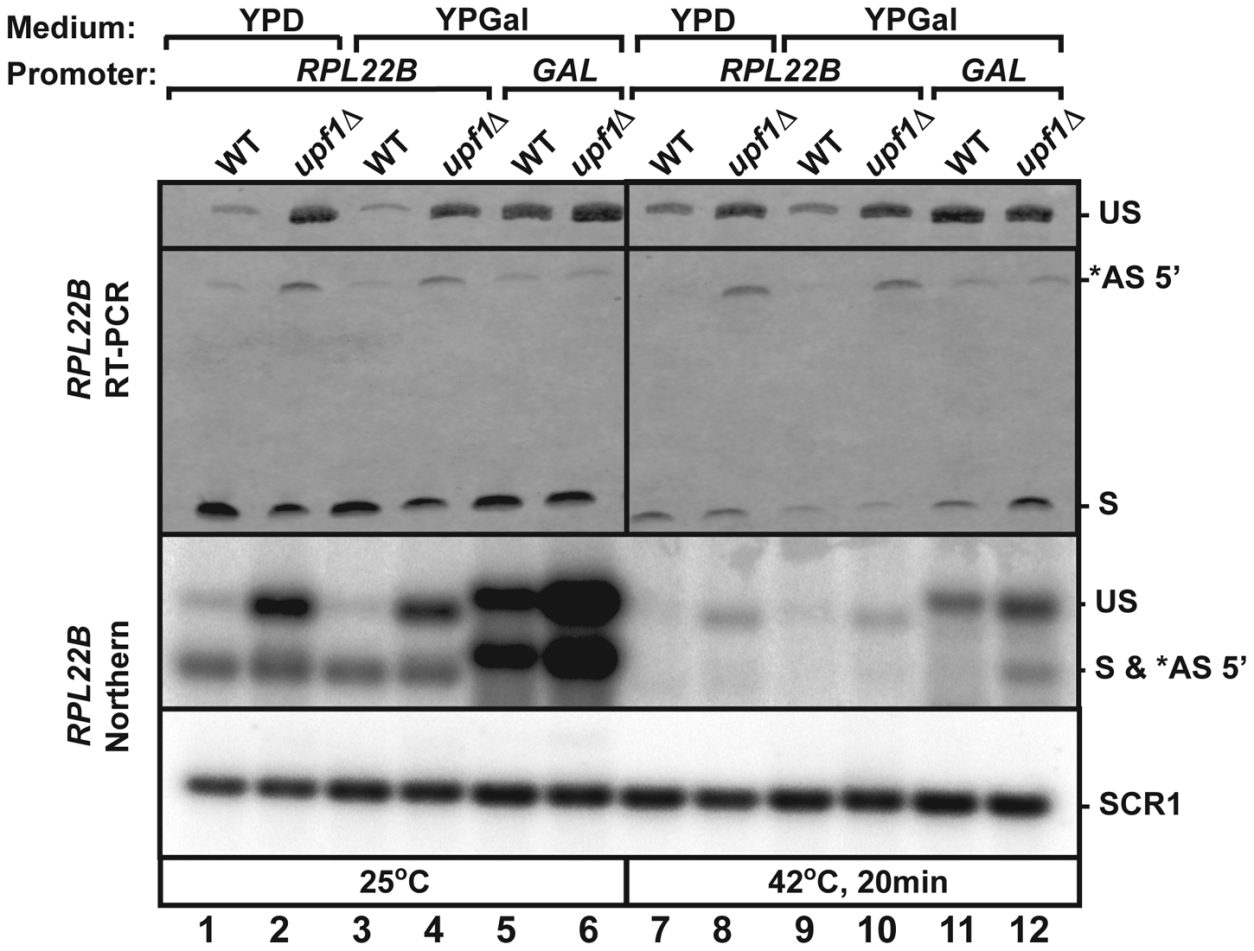
Replacement of *RPL22B* promoter by the GAL promoter results in a decrease in alternative 5′-splice site usage. Shown are the products generated when growing the indicated strains (wild-type or *upf1Δ* that contained the natural *RPL22B* promoter or the *GAL* promoter upstream *RPL22B*) in glucose (YPD) or galactose (YPGal)-containing media. Top panel, RT-PCR analysis. US, *AS 5′, and S indicate the location of the products corresponding to the unspliced, alternatively spliced and normal spliced products, respectively. Bottom Panel, Northern blot analysis. The labeling of the different species is similar to that of the top panel. SCR1 was used as a loading control.

Ribosomal protein genes are known to be transcriptionally regulated in stress conditions. To investigate the use of *RPL22B* 5′-SS selection independently from transcriptional inhibition under heat shock, we replaced the natural *RPL22B* promoter with a galactose-inducible promoter. The wild-type and *upf1* ***Δ*** strains containing the natural *RPL22B* promoter showed no detectable difference in *RPL22B* splicing patterns or expression when grown in galactose containing medium (YPGal) compared to glucose-containing medium (YPD) at 25°C (Fig. 6 lanes 1–4), either by RT-PCR (top panel) or northern blot (bottom panel). Strikingly, replacement of the normal *RPL22B* promoter by the *GAL* promoter resulted in an increase in overall *RPL22B* transcript levels, but also in a decrease in the use of the alternative 5′-SS (Fig. 6). The fact that the usage of the alternative splice site of *RPL22B* is reduced in this strain while transcript levels are higher overall argues against the hypothesis that alternative splice site usage is the result of splicing errors occurring at low frequencies, as if this were the case, one would expect higher levels of alternatively spliced *RPL22B* transcripts upon its overexpression in the strain in which the natural *RPL22B* promoter was swapped for the *GAL* promoter. Under heat shock conditions, the use of the alternative splice site was reduced 8.1 fold in the *upf1* ***Δ*** strain expressing *RPL22B* under the control of the *GAL* promoter compared to the *upf1* ***Δ*** strain expressing *RPL22B* from its natural promoter and grown in galactose medium (Fig. 6, lanes 10 and 12). Thus, alternative splicing regulation of *RPL22B* upon heat shock is tightly linked to the identity of the *RPL22B* promoter, as switching the identity of the promoter is sufficient to favor the use of the normal 5′-splice site. The mechanism by which the identity of the promoter influences alternative splice site selection is unclear, but could be linked to the influence of the promoter on the speed of transcription. Nevertheless, we can conclude from these results that transcriptional down-regulation and the increased use of the alternative 5′-SS provide synergistic mechanisms to limit the expression of *RPL22B* during stress, consistent with the global down-regulation of ribosome biogenesis during stress conditions.

## Discussion

### A significant fraction of splicing events in *S. cerevisiae* generates non-functional RNA or protein products

In this study we show that the ensemble of transcripts generated by splicing from the *S. cerevisiae* genome is highly complex. Most of the splicing events that we have characterized in this study are non-productive, either because they result in transcripts that are targeted by NMD, or because the protein products generated from these transcripts are predicted to be non-functional (e.g. *GPI15*). The large number of additional splice sites identified, and their relaxed conservation (Fig. 1D) imply that the rules governing splice selection are intrinsically more flexible than previously thought. This is further illustrated by the finding that a non-canonical AUG sequence in *GCR1* can be used as a 3′-SS in the absence of Prp18p (Fig. 3). In some cases, non-productive alternatively spliced transcripts accumulate only at low levels (e.g. *GCR1*, *GPI15*, Fig. 3). However, for other genes such as *TFC3*, the alternatively spliced non-productive transcripts represent a significant fraction (close to 30%) of all RNAs generated from this locus. Thus, non-productive splicing can significantly limit the expression of these genes. This was further demonstrated by mutagenesis of the non-productive splice site of *RPL22B*, as changing this site to a consensus sequence was sufficient to increase the splicing efficiency and the expression of this gene (Fig. 5B). Thus, the presence of alternative and sometimes sub-optimal splice sites that compete with the normal splice site contributes to an overall decrease in the amount of productively-spliced transcripts. Because the overlap in the alternative splicing events detected in all three NMD-deficient strains was limited (Fig. 1C), and because we detected by RT-PCR some alternative splicing events that escaped detection by RNA-Seq (e.g. *GCR1*), it is likely that we have not exhaustively identified the ensemble of splice sites that can be used by *S. cerevisiae*, and that additional splice sites will be identified by deeper sequencing or systematic RT-PCR analysis.

### Contribution of splicing factors to alternative splice site selection and splice site fidelity

The analysis of double mutants in which splicing factor mutations were combined with NMD inactivation revealed some important and unexpected functions for these factors on alternative splice site selection. We found that the Nam8p and Mud1p components are important for the selection of some, but not all of the alternative 5′-splice sites described here. In the case of *RPL22B*, this requirement was likely due to the fact that the alternative 5′-SS possesses a suboptimal splicing sequence, and therefore exhibits a weaker affinity for U1 binding, and a stronger requirement for Mud1p and Nam8p that impact the efficiency of U1 snRNP assembly on the alternative splice site. Strikingly, we identified a new role for Prp17p in favoring the use of upstream, branchpoint-proximal 3′-SS. In all cases that we have analyzed, Prp17p inactivation resulted in an increase in the use of the downstream 3′-SS. The mechanistic basis for this novel function that we describe here for Prp17p in promoting branchpoint proximal 3′-SS is not fully understood. Because 3′-SS close to the branchpoint are often the first ones that are being used, this novel function for Prp17p could be linked to promoting the ability of the spliceosome to scan and recognize 3′-SS close to the branchpoint, or to unwind secondary structures that mask branchpoint-proximal 3′-SS. The absence of Prp17p would result in a higher rate of misrecognition of 3′-SS and in the use of more distal 3′-SS. In addition, we found that the absence of Prp18p resulted in the selection of a non-canonical AUG 3′-SS in *GCR1*, and that this atypical 3′-SS was utilized to a greater extent during heat-shock, revealing a unique function for Prp18p in suppressing usage of a non-canonical 3′-SS. This function for Prp18p is independent from Prp22p's function in proofreading 3′-SS [41], but might complement its role to ensure the overall proper fidelity of 3′-SS selection. While we have demonstrated this function for *GCR1* only, a full genomic analysis of 3′-SS usage in the absence of Prp18p might reveal further examples of non-canonical 3′-SS being used.

### Spliceosome errors or bona-fide regulations?

The widespread occurrence of non-productive splice site usage described in this study begs the question of whether the use of these splice sites is the result of mistakes by the spliceosome, which occur at low frequency (as one might suggest based on their weaker consensus sequences) or whether they correspond to sites that have been selected throughout evolution for regulatory purposes. The sequence of some of these intronic, non-productive splice sites is conserved across closely related yeast species (Fig. S8 and S9), which, given the low conservation of intronic sequences in general, argues that this might reflect some degree of functional relevance. In addition, there is no obvious correlation between transcript levels and the occurrence of alternative splicing events (Fig. 1D), which argues against the suggestion that most of the alternative splicing events that we have mapped arise from low fidelity splicing events or errors that occur randomly, and which would be expected to be more frequently detected in highly abundant transcripts. Also, replacement of the *RPL22B* gene promoter results in higher transcript levels but reduces the usage of the alternative 5′-splice site of *RPL22B* (Fig. 6), providing another independent argument to suggest that the level of usage of alternative splice sites is not solely a reflection of overall transcript abundance. Finally, we show that the use of some of these alternative splice sites can be up-regulated during stress conditions (*RPL22B*, *GCR1*), and that this increased use participates in the down-regulation of *RPL22B* in stress conditions. Thus, this phylogenetically conserved, alternative, non-productive 5′-SS of *RPL22B* is functionally important because it contributes to the down-regulation of *RPL22B* during stress. This is shown by the fact that changing this sequence to a consensus sequence results in a significant increase in transcript levels upon NMD inactivation during stress (Fig. 5). The transcriptional down-regulation of ribosomal proteins during stress has been documented previously [45]. We show here that the promoter of the *RPL22B* gene is essential not only because it drives transcriptional repression during stress, but also because it controls the switch in 5′-SS selection that contributes to the overall repression of *RPL22B* during heat-shock. Thus, a combination of transcriptional and post-transcriptional regulations, through splicing inhibition [43], [44], degradation of unspliced RNAs by NMD [12], [47] and use of non-productive splice sites (this study) contributes to the repression of ribosomal protein production during stress. While several non-RPG transcripts analyzed in these stress conditions did not shown any changes, *GCR1* did exhibit a change in the use of alternative splice sites during stress (Fig. 4B). This result raises the possibility that other intron-containing genes may be regulated similarly by alternative splicing as a function of different environmental growth conditions. Overall our study has revealed that the pattern of splicing events in the model eukaryote *S. cerevisiae* is highly complex, but masked by NMD-mediated degradation. Given the recent report that another single cell eukaryote, *S.pombe* shows alternative splicing patterns conserved in higher eukaryotes [50], these observations suggest that alternative splicing provides an important contribution to genetic regulations and adaptations to environmental changes in unicellular eukaryotes. However, in the case of *S.cerevisiae*, the use of alternative splice sites have evolved towards fine tuning transcript levels, rather than generating proteome diversity as shown in higher eukaryotes.

## Materials and Methods

### Yeast culture and RNA analysis

Yeast strains were grown at 25°C in YPD medium, unless indicated otherwise in the figures. For heat shock treatment, strains were pre-grown in YPD at 25°C, spun down in 50 mL Falcon tubes, resuspended in pre-warmed YPD medium and heat shocked for 20 min before harvesting. For LiCl treatment, yeast strains were grown to mid-log phase in YPD rich media at 30°C, harvested by centrifugation in 50 mL Falcon tubes, washed once with pre-warmed 50 mL of YPD+300 mM LiCl before being resuspended in pre-warmed YPD with 300 mM LiCl for 10 minutes. For Rapamycin treatment, cells were grown to mid-log phase in rich media (at 30°C), and rapamycin from a stock solution of 1 mg/mL in 90% ethanol, 10% Tween-20 was added to a final concentration of 200 ng/mL and cells were incubated for 20 minutes. The same volume of 90% ethanol, 10% Tween-20 solution used for the rapamycin treatment was added to the negative control. Sample preparation and RNA sequencing was performed by Illumina. RT-PCR analysis and northern blot was performed as described [21].

### Mapping reads

High throughput sequencing data have been deposited in the GEO database (accession GSE55213). All sequence files were aligned against the 2008 SGD assembly of the *Saccharomyces cerevisiae* genome. The novoalign software package (www.novocraft.com) and the BLAT alignment tool [22] were used to align 75 base pair reads in two steps. In the first step, sequences were aligned with novoalign allowing for up to four mismatches and no gaps. In the second step, sequences that failed to align in the first step were aligned with BLAT allowing three mismatches and gaps up to 20000 nucleotides in length. A sequence was kept for further analysis if it mapped with equal score to at most two genomic locations and did not contain a gap smaller than ten nucleotides.

### Intronic sequences counts

Intronic sequence expression representative of unspliced RNAs was quantified for each ICG by summing reads that aligned to introns and exon-intron boundaries. Values between samples were normalized by total mapped reads to account for lane effects. p-values were computed by modeling each ICG wild-type count as a poisson random variable and calculating the probability of observing each mutant count if it were drawn from the same distribution.

### Quantification of alternative splicing events

Alternative splicing events were defined as splicing events that are within ICGs and are supported by sequencing but that are not annotated in the *Saccharomyces* Genome Database (SGD). Counts of total alternative splicing events and PTC-generating alternative splicing events were quantified by summing all unique alternative splicing events in each sample. To determine if an alternative splicing event is PTC-generating we constructed the splice product's sequence using the novel splicing event in the otherwise canonical transcript sequence. Counts between samples were normalized by sequencing depth. p-values were calculated by modeling the wild-type count as a poisson random variable and calculating the probability of observing each mutant's count for both total alternative splicing events and PTC-generating alternative splicing events. Venn diagrams of agreement between samples were generated using BioVenn [51].

### Splice site consensus sequence

Consensus sequences for 5′ and 3′ ends of both canonical splice sites and alternative splice sites were represented as sequence logos. Sequence logos were constructed using the MATLAB (MathWorks) seqlogo function.

## Supporting Information

Figure S1 Quantification of the SRC1-L and SRC1-S isoforms in wild-type, *upf1Δ* and splicing mutants. Shown is the percentage of the *SRC1-L* and *SRC1-S* transcripts in various strains. Values shown are the average and standard deviations obtained from RT-PCR experiments of three independent cultures for each strain.
(TIF)

Figure S2 Quantification of the usage of the normal and alternative 5′-splice sites of *RPL22B* in wild-type, *upf1Δ* and splicing mutants. Shown is the percentage of transcripts spliced at the normal 5′-splice site (red) and at the alternative 5′-splice site (blue). Values shown are the average and standard deviations obtained from RT-PCR experiments of three independent cultures for each strain.
(TIF)

Figure S3 Quantification of the usage of the normal and alternative 3′-splice sites of *TFC3* in wild-type, *upf1Δ* and splicing mutants. Shown is the percentage of transcripts spliced at the normal 3′-splice site (blue) and at the alternative 5′-splice site (red). Values shown are the average and standard deviations obtained from RT-PCR experiments of three independent cultures for each strain.
(TIF)

Figure S4 Quantification of the usage of the two alternative 3′-splice sites of *TAN1* in wild-type, *upf1Δ* and splicing mutants. Shown is the percentage of transcripts spliced at the alternative 3′-splice site #1 (blue) or #2 (red) compared to all the spliced transcripts. Values shown are the average and standard deviations obtained from RT-PCR experiments of three independent cultures for each strain.
(TIF)

Figure S5 Validation of the use of the AUG alternative 3′ splice site of *GCR1* by RT-PCR. Sequencing of the cloned *D and *E cDNAs determined the location of the splice junction, while sequencing of unspliced cDNAs was used to confirm that this unusual alternative 3′-SS was indeed AUG, and not a SNP or other mutation of the *GCR1* gene that would have converted it into an AAG. RT-PCR confirmation of the use of this AUG 3′-SS was performed using reverse primers spanning the splice junction to specifically amplify distinct splicing events; either associated with *D, *E, or unspliced. The use of the AUG 3′ SS was also confirmed using an intronic reverse primer just downstream of the AUG sequence and detected *D, *E, and unspliced products, as predicted (Fig. S1). A. RT-PCR strategy. All PCR include the same forward primer For, and various reverse primers that hybridize to the indicated regions of *GCR1*. B. RT-PCR data. Shown are the PCR products obtained from the different reverse primers shown in A.
(TIF)

Figure S6 Quantification of the abundance of the major alternatively spliced forms of *GCR1* in wild-type, *upf1Δ* and splicing mutants. Shown is the percentage of the *D (blue), *A (red) or *C (green) spliced forms. Values shown are the average and standard deviations obtained from RT-PCR experiments of three independent cultures for each strain.
(TIF)

Figure S7 RT-PCR analysis of GCR1 splicing in the *prp18* and *prp22-1* mutant strains. The identity of the different spliced products is labeled according to Figure 2.
(TIF)

Figure S8 Conservation of the intronic alternative 5′-SS in *RPL22B*. **A**. Screen capture of the web browser showing the RNA-Seq reads mapped for *RPL22B* that use the alternative 5′-SS in black, and the sequence conservation in closely related yeast species as blue peaks. The peak showing conservation of the intronic alternative 5′splice site is shown on the right, since the gene is encoded on the Crick strand. **B**. Zoomed in view of the conservation of the sequence of the alternative 5′-SS (ACAAC sequence because of the Crick Strand).
(PDF)

Figure S9 Conservation of the intronic alternative 3′-SS in *TAN1*. **A**. Screen capture of the web browser showing the RNA-Seq reads mapped for *TAN1* that use the intronic alternative 3′-SS in black, and the sequence conservation in closely related yeast species as blue peaks. **B**. Zoomed in view of the conservation of the sequence of the alternative intronic AAG 3′-SS.
(PDF)

Figure S10 Quantification of the usage of the alternative 5′-splice sites of *RPL22B* in various normal media (YPD, SDC) or in stress conditions (Heat shock, amino acid starvation). Values shown are the average and standard deviations obtained from RT-PCR experiments of three independent cultures for each strain.
(TIF)

Figure S11 RT-PCR analysis of the spliced products of *TAN1* and *TFC3* under stress conditions. Shown are the products for the unspliced (US), normal spliced product (S), and the alternatively spliced species (AS) described in Figure 2.
(TIF)

Figure S12 Quantitation of the use of the alternative 5′-splice site of *RPL22B* under normal growth conditions (25°C) and after a 20 min heat shock at 42°C. Plotted are the amount of transcript spliced at the alternative splice site divided by the values obtained for all spliced species for the indicated strains. Shown are the average of 4 to 5 independent experiments with the standard deviations.
(TIF)

Figure S13 Quantitation of the use of the normal and alternative 5′-splice site of *RPL22B* under normal growth conditions in minimal medium (SDC) and after amino acid starvation (-AA) for the strains expressing the natural (N) GUUUGU sequence at the alternative 5′ splice site of *RPL22B*, or the consensus (CS) GUAUGU sequence in the context of wild-type *UPF1* (WT) or when *UPF1* has been deleted (**Δ**). Plotted are the amount of transcript spliced at the normal and alternative splice sites divided by the values obtained for all spliced species. Shown are the average of 3 independent experiments with the standard deviations.
(TIF)

Table S1 Statistics of RNA-Seq analysis sequence alignments.
(XLSX)

Table S2 Number of alternative splicing events detected in wild-type and NMD-deficient strains.
(XLSX)

Table S3 Mapping of RNA-Seq reads in wild-type and NMD-deficient strains in various genomic elements and in intron-containing genes.
(XLSX)

Table S4 List of intron-containing genes for which no alternative splicing events were detected. Shown is the list of intron-containing genes for which no alternative splicing junctions were detected in any of the strains sequenced. The adjusted number of reads obtained from each strain (RPKM) and the sequence of the 5′- and 3′-splice sites is shown for each of these genes.
(XLSX)

Table S5 List of intron-containing genes for which alternative splicing events were detected. Shown is the list of intron-containing genes for which alternative splicing junctions were detected in at least one of the strains sequenced. Shown is the number of splice junction sequences counts for the normal (blue) and alternative (green) splicing events. For the alternative splicing events, the 5′ and/or 3′ splice sites which differ from the normal splice sites are marked by an asterisk. The position of these splice sites on each chromosome is also indicated. The adjusted number of reads obtained from each strain (RPKM) and the sequence of the normal and alternative 5′- and 3′-splice sites is shown for each of these genes.
(XLSX)

Table S6 List of alternative proteins potentially generated by alternative splicing in wild-type or NMD mutants. For each open reading frame, a portion of the normal protein sequence is shown on the first line, and the sequence that differs upon the alternative splicing event is shown below. Amino acids maintained between the two forms are indicated in red. Amino acids that differ between the two forms are highlighted in bold and black. In the case of splicing events inducing a deletion, a delta sign has been added with a number corresponding to the number of amino acid deleted. The numbers of the first and last amino acids shown is indicated before and after each protein sequence, respectively. The numbers in brackets that follow each protein sequence correspond to the number of reads for the splice junctions in the wild-type strain, *upf1* ***Δ***, *upf2* ***Δ*** and *upf3* ***Δ*** mutants.
(DOCX)
