# Supplementary figures and images for: Widespread Use of Non-productive Alternative Splice Sites in Saccharomyces cerevisiae
Source: PLoS Genet. 2014 Apr 10;10(4):e1004249. doi: 10.1371/journal.pgen.1004249 (PMC3983031; doi:10.1371/journal.pgen.1004249)

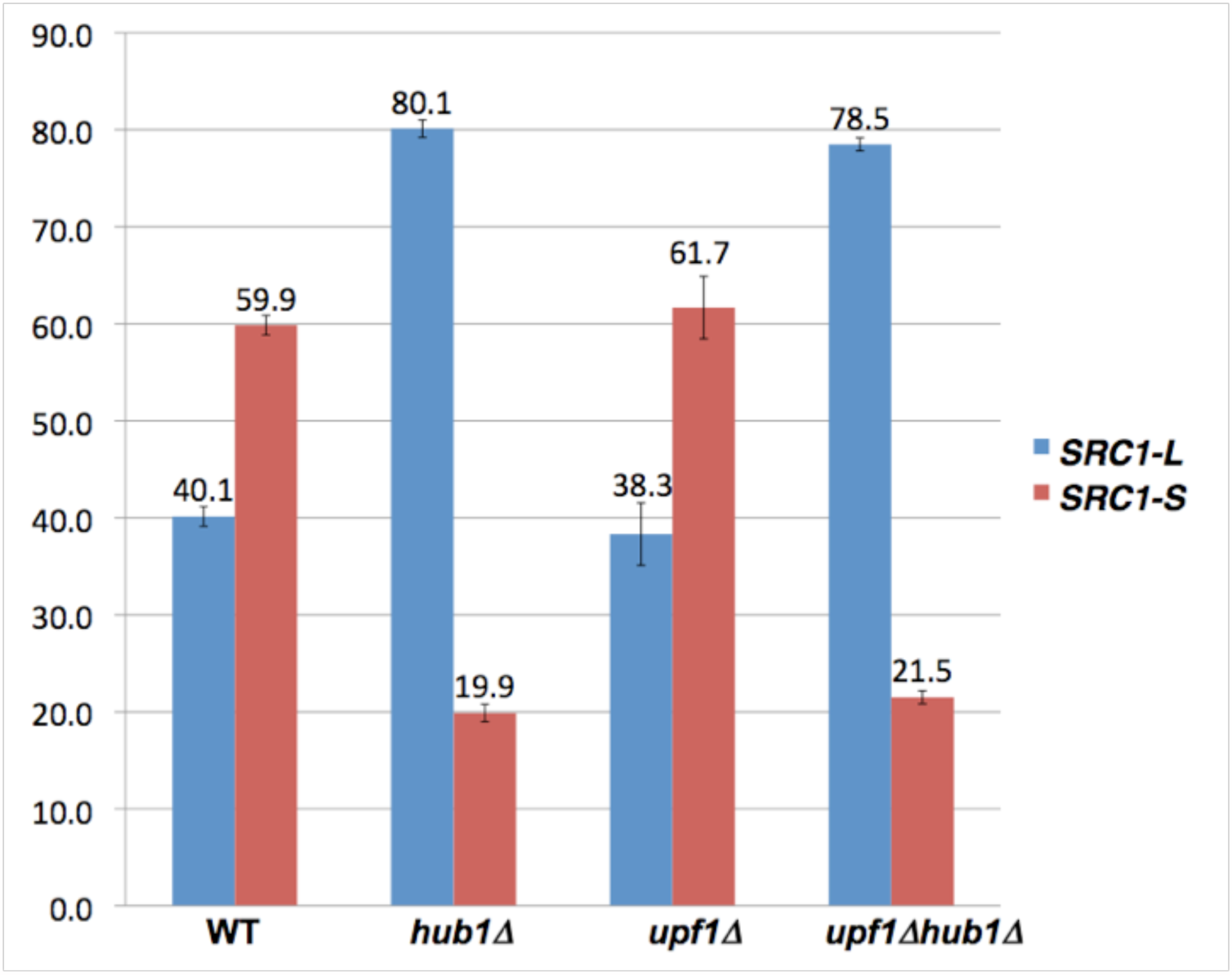

Supplement: Figure S1 — Quantification of the SRC1-L and SRC1-S isoforms in wild-type, upf1Δ and splicing mutants. Shown is the percentage of the SRC1-L and SRC1-S transcripts in various strains. Values shown are the average and standard deviations obtained from RT-PCR experiments of three independent cultures for each strain. (TIF) [file pgen.1004249.s001.tif]

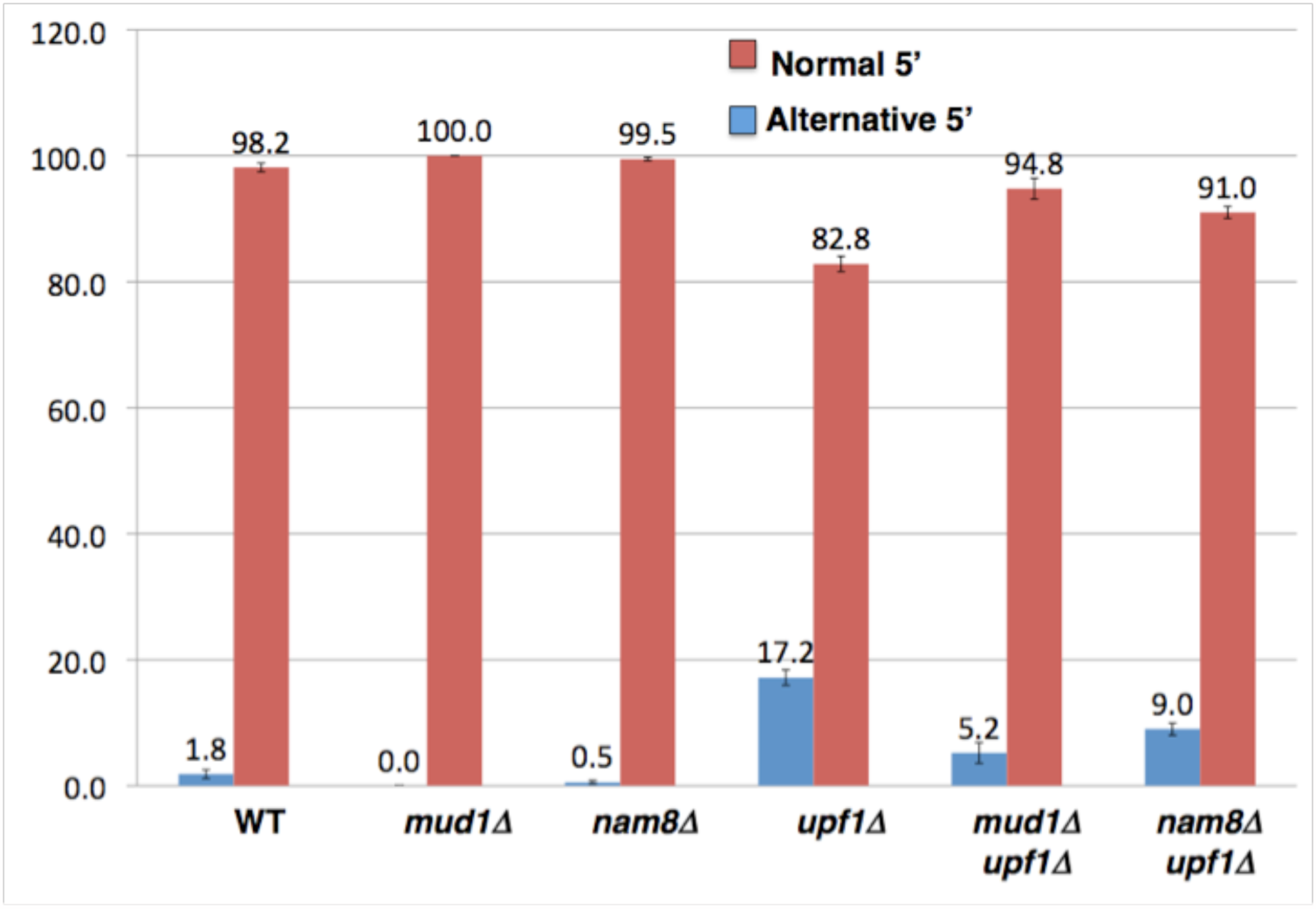

Supplement: Figure S2 — Quantification of the usage of the normal and alternative 5′-splice sites of RPL22B in wild-type, upf1Δ and splicing mutants. Shown is the percentage of transcripts spliced at the normal 5′-splice site (red) and at the alternative 5′-splice site (blue). Values shown are the average and standard deviations obtained from RT-PCR experiments of three independent cultures for each strain. (TIF) [file pgen.1004249.s002.tif]

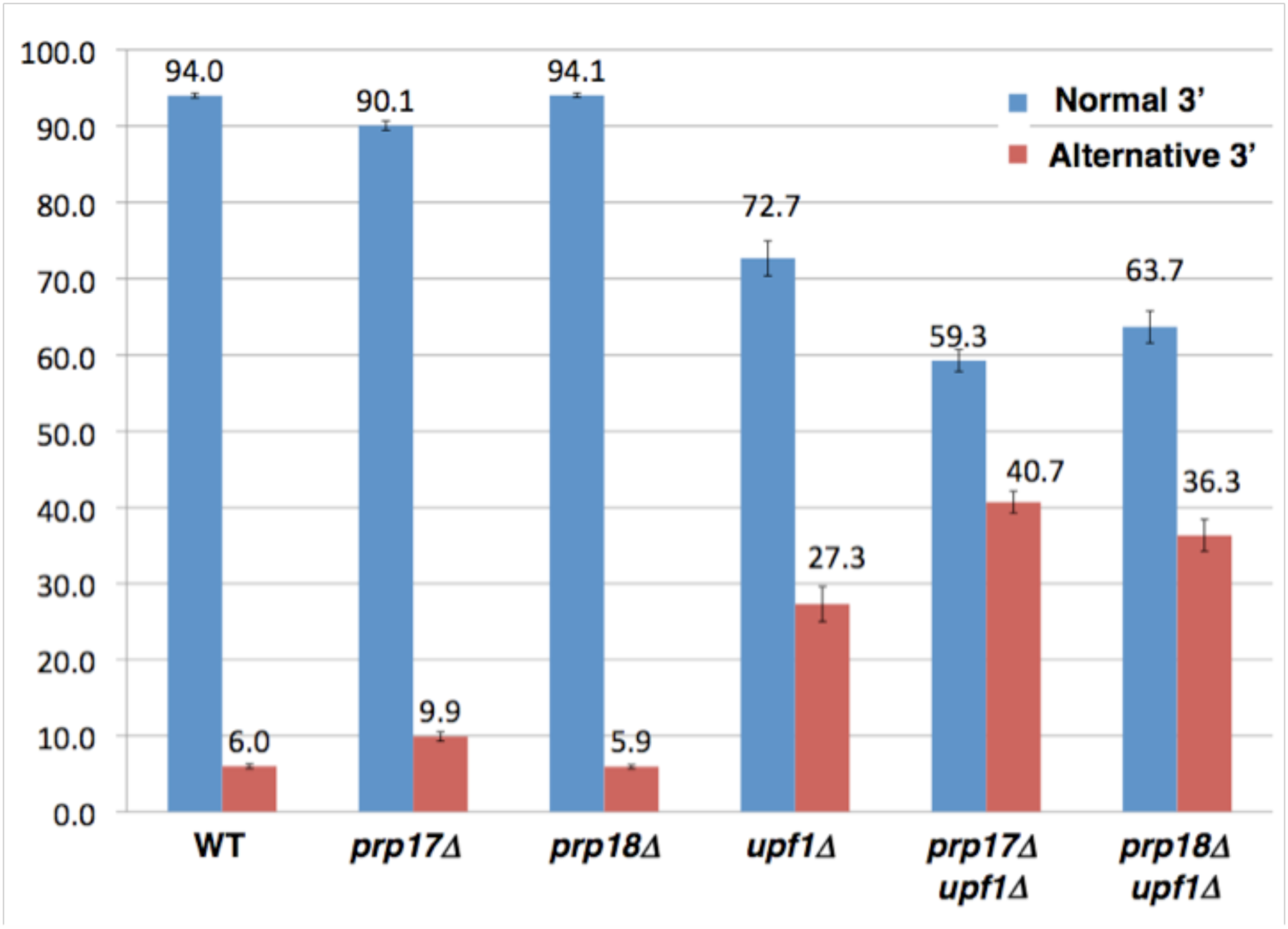

Supplement: Figure S3 — Quantification of the usage of the normal and alternative 3′-splice sites of TFC3 in wild-type, upf1Δ and splicing mutants. Shown is the percentage of transcripts spliced at the normal 3′-splice site (blue) and at the alternative 5′-splice site (red). Values shown are the average and standard deviations obtained from RT-PCR experiments of three independent cultures for each strain. (TIF) [file pgen.1004249.s003.tif]

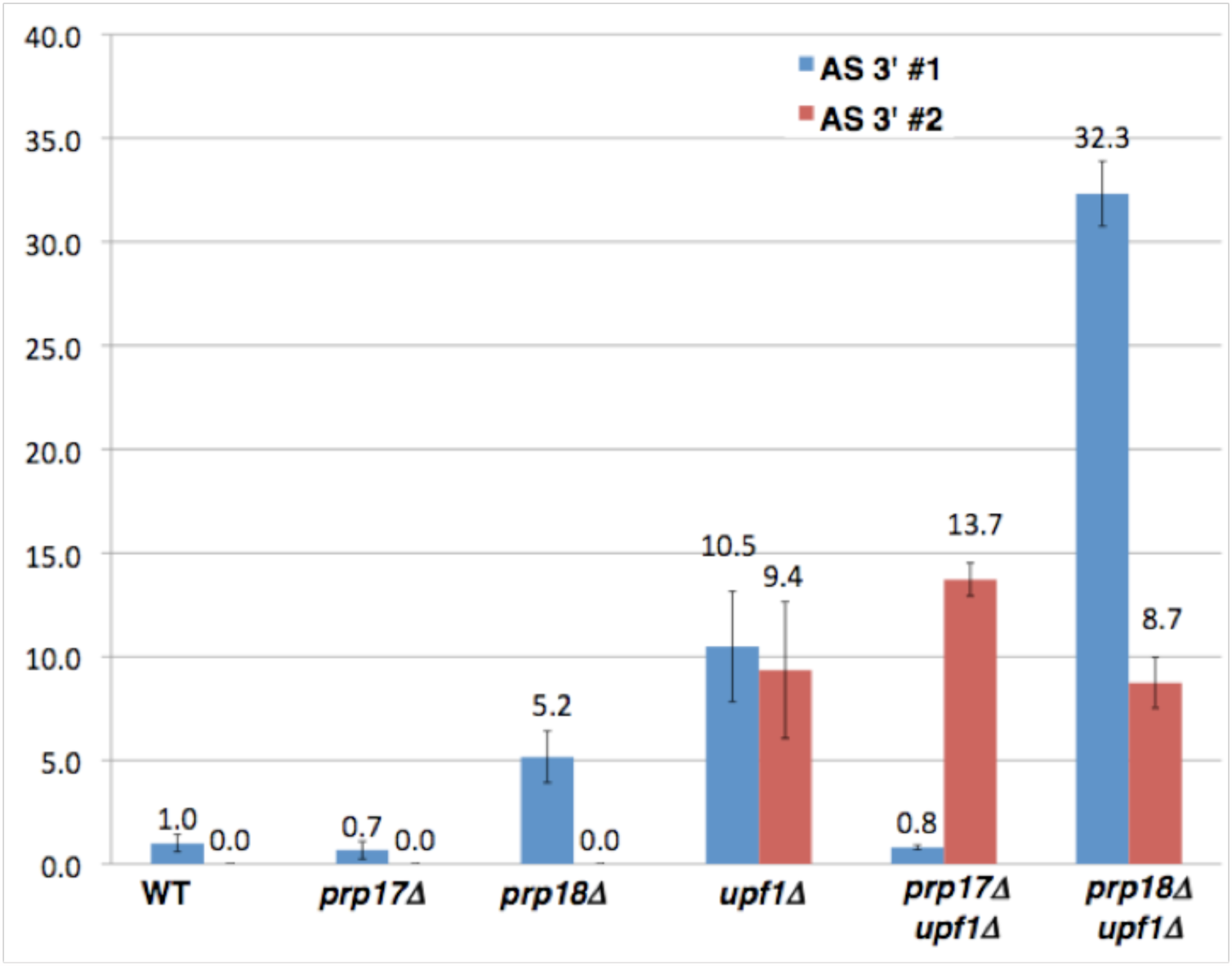

Supplement: Figure S4 — Quantification of the usage of the two alternative 3′-splice sites of TAN1 in wild-type, upf1Δ and splicing mutants. Shown is the percentage of transcripts spliced at the alternative 3′-splice site #1 (blue) or #2 (red) compared to all the spliced transcripts. Values shown are the average and standard deviations obtained from RT-PCR experiments of three independent cultures for each strain. (TIF) [file pgen.1004249.s004.tif]

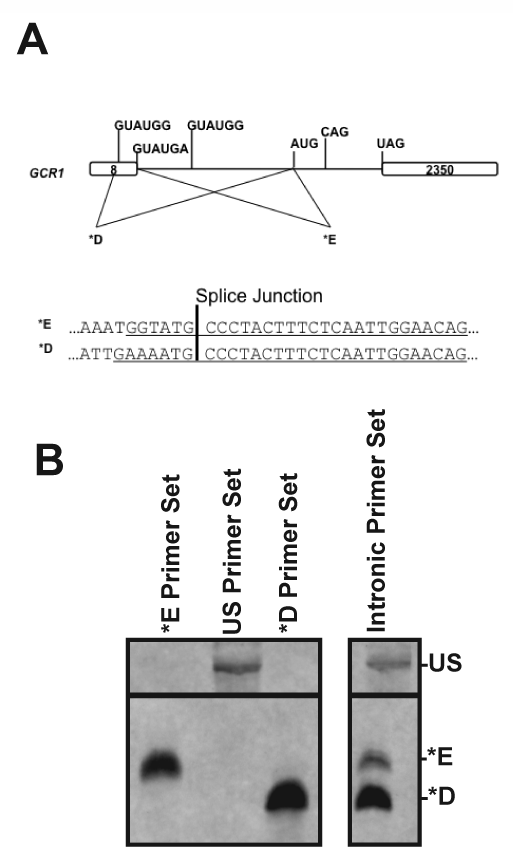

Supplement: Figure S5 — Validation of the use of the AUG alternative 3′ splice site of GCR1 by RT-PCR. Sequencing of the cloned *D and *E cDNAs determined the location of the splice junction, while sequencing of unspliced cDNAs was used to confirm that this unusual alternative 3′-SS was indeed AUG, and not a SNP or other mutation of the GCR1 gene that would have converted it into an AAG. RT-PCR confirmation of the use of this AUG 3′-SS was performed using reverse primers spanning the splice junction to specifically amplify distinct splicing events; either associated with *D, *E, or unspliced. The use of the AUG 3′ SS was also confirmed using an intronic reverse primer just downstream of the AUG sequence and detected *D, *E, and unspliced products, as predicted (Fig. S1). A. RT-PCR strategy. All PCR include the same forward primer For, and various reverse primers that hybridize to the indicated regions of GCR1. B. RT-PCR data. Shown are the PCR products obtained from the different reverse primers shown in A. (TIF) [file pgen.1004249.s005.tif]

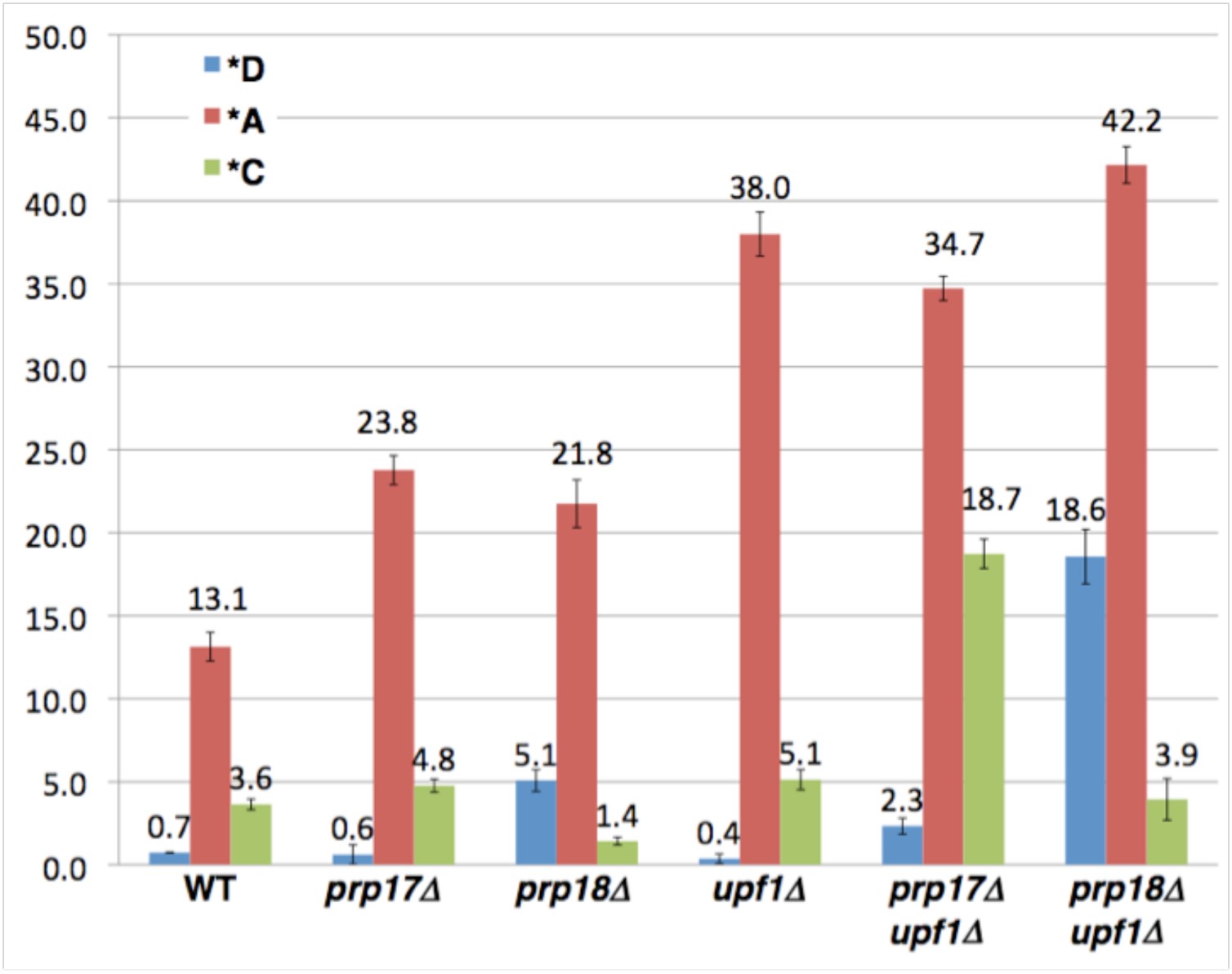

Supplement: Figure S6 — Quantification of the abundance of the major alternatively spliced forms of GCR1 in wild-type, upf1Δ and splicing mutants. Shown is the percentage of the *D (blue), *A (red) or *C (green) spliced forms. Values shown are the average and standard deviations obtained from RT-PCR experiments of three independent cultures for each strain. (TIF) [file pgen.1004249.s006.tif]

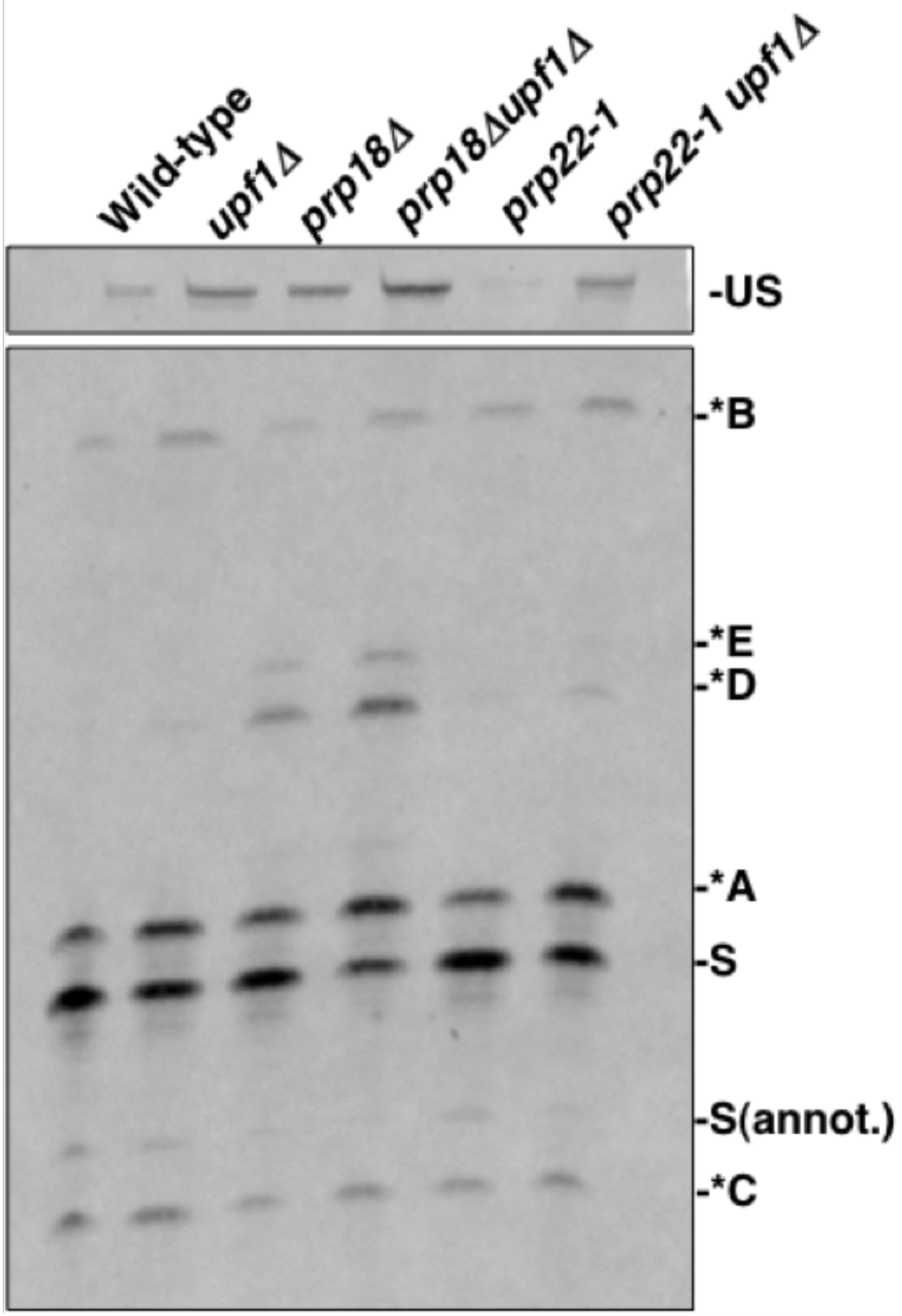

Supplement: Figure S7 — RT-PCR analysis of GCR1 splicing in the prp18 and prp22-1 mutant strains. The identity of the different spliced products is labeled according to Figure 2. (TIF) [file pgen.1004249.s007.tif]

**A**

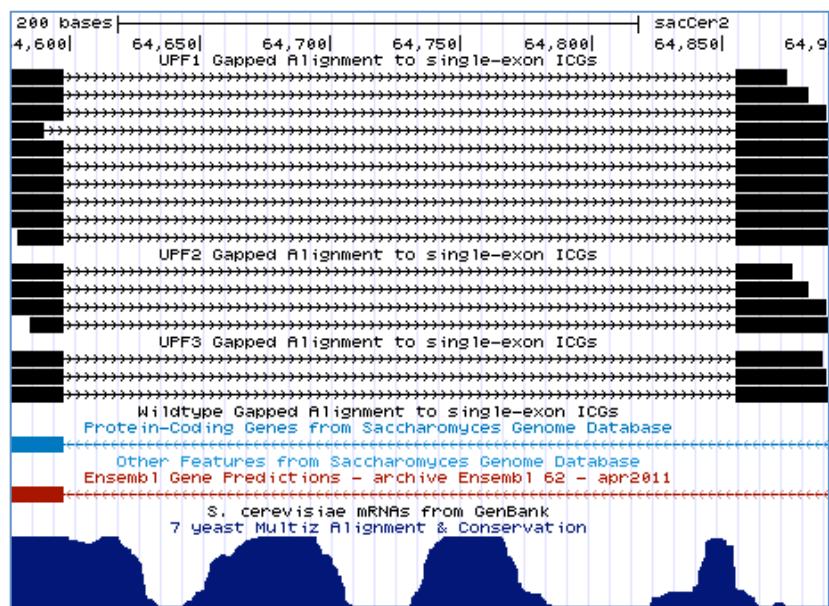

**B**

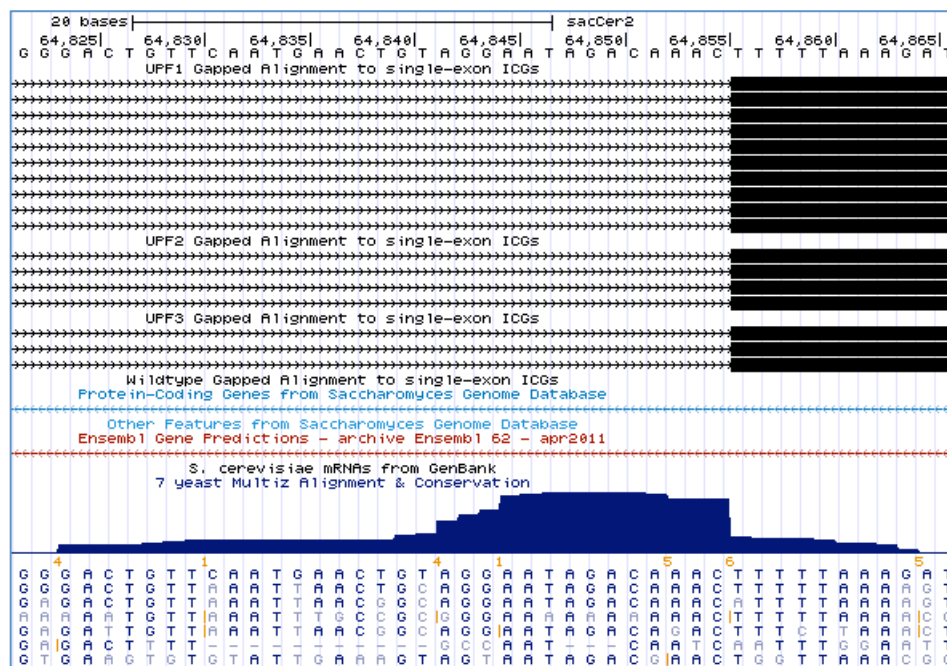

Supplement: Figure S8 — Conservation of the intronic alternative 5′-SS in RPL22B. A. Screen capture of the web browser showing the RNA-Seq reads mapped for RPL22B that use the alternative 5′-SS in black, and the sequence conservation in closely related yeast species as blue peaks. The peak showing conservation of the intronic alternative 5′splice site is shown on the right, since the gene is encoded on the Crick strand. B. Zoomed in view of the conservation of the sequence of the alternative 5′-SS (ACAAC sequence because of the Crick Strand). (PDF) [file pgen.1004249.s008.pdf]

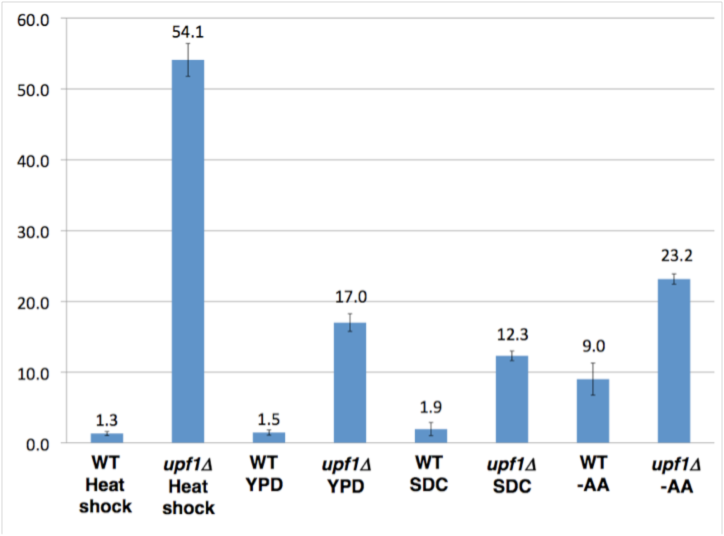

Supplement: Figure S10 — Quantification of the usage of the alternative 5′-splice sites of RPL22B in various normal media (YPD, SDC) or in stress conditions (Heat shock, amino acid starvation). Values shown are the average and standard deviations obtained from RT-PCR experiments of three independent cultures for each strain. (TIF) [file pgen.1004249.s010.tif]

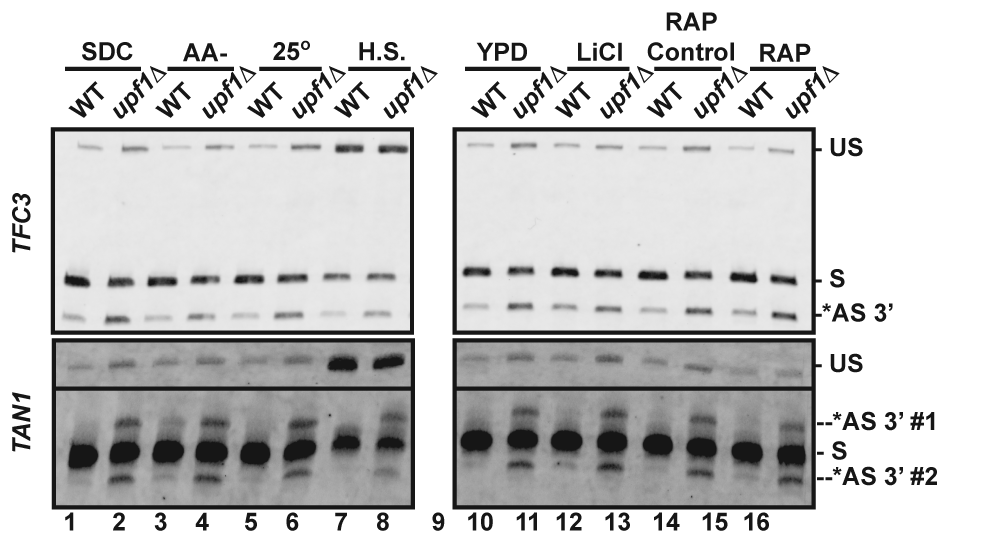

Supplement: Figure S11 — RT-PCR analysis of the spliced products of TAN1 and TFC3 under stress conditions. Shown are the products for the unspliced (US), normal spliced product (S), and the alternatively spliced species (AS) described in Figure 2. (TIF) [file pgen.1004249.s011.tif]

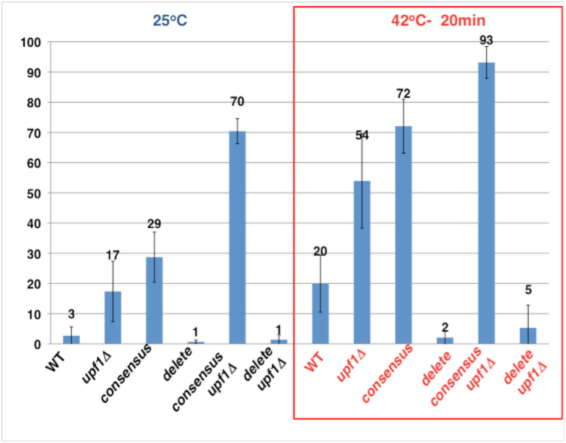

Supplement: Figure S12 — Quantitation of the use of the alternative 5′-splice site of RPL22B under normal growth conditions (25°C) and after a 20 min heat shock at 42°C. Plotted are the amount of transcript spliced at the alternative splice site divided by the values obtained for all spliced species for the indicated strains. Shown are the average of 4 to 5 independent experiments with the standard deviations. (TIF) [file pgen.1004249.s012.tif]

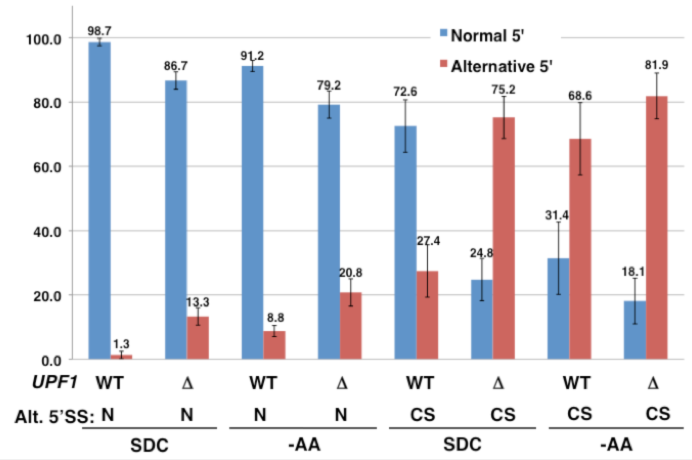

Supplement: Figure S13 — Quantitation of the use of the normal and alternative 5′-splice site of RPL22B under normal growth conditions in minimal medium (SDC) and after amino acid starvation (-AA) for the strains expressing the natural (N) GUUUGU sequence at the alternative 5′ splice site of RPL22B, or the consensus (CS) GUAUGU sequence in the context of wild-type UPF1 (WT) or when UPF1 has been deleted (Δ). Plotted are the amount of transcript spliced at the normal and alternative splice sites divided by the values obtained for all spliced species. Shown are the average of 3 independent experiments with the standard deviations. (TIF) [file pgen.1004249.s013.tif]
