# Supplementary material for: Widespread Use of Non-productive Alternative Splice Sites in Saccharomyces cerevisiae
Source: PLoS Genet. 2014 Apr 10;10(4):e1004249. doi: 10.1371/journal.pgen.1004249 (PMC3983031; doi:10.1371/journal.pgen.1004249)
Supplement: Table S6 — List of alternative proteins potentially generated by alternative splicing in wild-type or NMD mutants. For each open reading frame, a portion of the normal protein sequence is shown on the first line, and the sequence that differs upon the alternative splicing event is shown below. Amino acids maintained between the two forms are indicated in red. Amino acids that differ between the two forms are highlighted in bold and black. In the case of splicing events inducing a deletion, a delta sign has been added with a number corresponding to the number of amino acid deleted. The numbers of the first and last amino acids shown is indicated before and after each protein sequence, respectively. The numbers in brackets that follow each protein sequence correspond to the number of reads for the splice junctions in the wild-type strain, upf1 Δ, upf2 Δ and upf3 Δ mutants. (DOCX) [file pgen.1004249.s019.docx]

**Table S6. List of alternative proteins potentially generated by alternative splicing in wild-type or NMD mutants.**

For each open reading frame, a portion of the normal protein sequence is shown on the first line, and the sequence that differs upon the alternative splicing event is shown below. Amino acids maintained between the two forms are indicated in red. Amino acids that differ between the two forms are highlighted in bold and black. In the case of splicing events inducing a deletion, a delta sign has been added with a number corresponding to the number of amino acid deleted. The numbers of the first and last amino acids shown is indicated before and after each protein sequence, respectively. The numbers in bracket that follow each protein sequence correspond to the number of reads for the splice junctions in the wild-type strain, *upf1Δ*, *upf2Δ* and *upf3Δ* mutants.

YAL001C:

17:KIASNKG**KITLNQL**WDISGKYF:38 [2 5 5 5]

17:KIASNKG**M**WDISGKYF:32 [0 0 1 4]

YBL111C:

1:MKVSDR**RKFEKANFDEFESALNNKNDLVHCPSITLFESIPTEVRSFYEDEKSGLIKVVKFRTGAMDRKRSFEKIVVSVMVGKNVQKFLTFVEDEPDFQGGPIPSKYLIPKKINLMVYTLFQVHTLKFNRKDYDTLSLFYLNRGYYNELSFRVLERCYEIASARPNDSSTMRTFTDFVSGTPIVRSLQKSTIRKYGYNLAPYMFLLLHVDELSIFSAYQASLPGEKKVDTERLKRDLCPRKPTEIKYFSQICNDMMNKKDRLGDVL**ATAQRIRRRY:275 [0 0 0 0]

1:MKVSDR**PLSR**ATAQRIRRRY:20 [0 0 0 1]

YBL087C:

9:TKFRIS**LGLPVGAIMNCADNS**GARNLY:35 [1102 886 1000 830]

9:TKFRIS**∆15**GARNLY:20 [0 1 0 1]

YBL050W:

4:PVELLK**RAEKKGVPSSGFMKLF**SGSDS:30 [48 49 41 46]

4:PVELLK**∆16**SGSDS:14 [0 3 0 2]

YBL040C:

1:MNPFRIL**∆1**GDLSHLT:14 [66 55 68 62]

1:MNPFRIL**A**GDLSHLT:15 [0 4 4 3]

YBR111W-A:

9:NYE**LISNELKARLLQEGWVDKVKDLTKSEMNINESTNFTQILSTVEPKAL**EMVS:74 [47 50 16 44]

9:NYE**∆47**EMVS:27 [0 1 0 1]

YBR255C-A:

15:VKQLTKE**ITVASCIGAAQGALFSIASALLLRRFSSVYR**NVRTQVRV:60 [7 10 16 13]

15:VKQLTKE**∆31**NVRTQVRV:29 [1 2 0 0]

YDL075W:

12:YTINLHK**R**LHGVSFK:26 [2419 2803 2685 2449]

12:YTINLHK**∆1**LHGVSFK:25 [0 1 0 0]

YDL029W:

3:PHNPI**VLDQ**GTGFVKI:18 [64 71 57 75]

3:PHNPI**∆4**GTGFVKI:14 [0 1 1 0]

YDR005C:

1:MK**FIDELDIERVNQTLNFETNDCKIVGSCDIFTTKAVASDRKLYKTIDQHLDTILQENENYNATLQQQLAAPETNQSPCSSPFYSNRRDSNSFWEQKRRISFS**EYNSNN:109 [20 51 46 47]

1:MK**∆101**EYNSNN:8 [0 1 0 0]

YDR005C:

1:MKF**IDELDIERVNQTLNFETNDCKIVGSCDIFTTKAVASDRKLYKTIDQHLDTILQENENYNATLQQQLAAPETNQSPCSSPFYSNRRDSNSFWEQKRRISFSEYNSNNNTNNSNGNSSNNNNYSGPNGSSPATFPKSAKLNDQNLKELVSNYDSGSMSSSSLDSSSKNDERIRRRSSSSISSF**KSGKSSN:191 [20 51 46 47]

1:MKF∆181KSGKSSN:10 [0 1 0 0]

YDR064W:

1:MG**RMHSA**GKGISS:13 [995 912 829 844]

1:MG**∆5**GKGISS:8 [0 0 1 0]

YDR064W:

1:MGR**MHSAGKGIS**SSAI:16 [995 912 829 844]

1:MGR**I**SSAI:8 [0 0 0 1]

YDR129C:

1:MNIVKLQ**RKFPILTQEDLFSTIEKFR**AIDLDDKGWV:36 [53 57 55 52]

1:MNIVKLQ**∆19**AIDLDDKGWV:17 [0 3 4 3]

YDR139C:

45:FQGKQI**DDKLTV**TDAHLVE:63 [43 71 100 98]

45:FQGKQI**∆6**TDAHLVE:57 [0 0 2 1]

YDR397C:

7:NVSLPK**A**TVQKM:18 [90 83 50 80]

7:NVSLPK**GMLVIL**TVQKM:23 [0 0 1 0]

YDR424C:

4:ENKST**PIVKASDIT**DKLKEDI:24 [23 14 16 24]

4:ENKST**R**DKLKEDI:16 [0 0 1 1]

YDR447C:

1:M**GR**VRTKTVKR:11 [141 152 136 142]

1:M**∆2**VRTKTVKR:9 [0 2 1 2]

YDR471W:

7:AGKVA**VVVRGRYAGKKVVIVKPHDEGSKSHPFGHALVAGIERYPSKVTKKHGAKKVAKRTKIKPFIKVVNYNHLLPTRYTLDVEAFKSVVSTETFEQPSQREEAKKVVKKA**FEERHQ:123 [289 175 304 220]

7:AGKVA**∆106**FEERHQ:17 [0 0 1 0]

YEL076C-A:

167:SSDGIS**DTLTVIQSFSYSLLPVLS**ATYTSM:195 [0 0 0 0]

167:SSDGIS**∆18**ATYTSM:178 [0 0 1 0]

YEL076C-A

163:VPSSDGIS**DTLTVIQSFSYSLLPVLSATYTSMIQQDASNCTLITTRTVHRSLD***:217 [0 0 0 0]

163:VPSSDGIS**RIQLYTDYNKNGSSEPRL**:189 [0 0 1 0]

YER056C-A:

7:FRRRNP**YNTRSNKIKVVKT**PGGILRA:32 [820 680 664 578]

7:FRRRNP**S**PGGILRA:20 [1 0 0 2]

YFL034C-A:

1:MAPN**TSR**KQKVI:12 [72 77 87 68]

1:MAPN**∆3**KQKVI:9 [0 1 2 2]

YGL030W:

1:M**∆1**APVKSQESINQ:12 [889 777 1107 834]

1:M**V**APVKSQESINQ:13 [2 7 5 4]

YGL030W:

1:M**∆5**APVKSQESINQ:12 [889 777 1107 834]

1:M**VRFQQ**APVKSQESINQ:17 [1 1 2 0]

YGR034W:

1:MAKQSL**∆5**DVSSDR:12 [943 908 1021 954]

1:MAKQSL**YLFSI**DVSSDR:17 [0 2 1 0]

YGR118W:

17:VHRRN**NRWAENNYKKRLLGTAFKSSPFGGSSHAKGIVLEKLGIESKQPNSAIRKCVRVQLIKNGKKVTAFVPNDGCLNFVDENDEVLLAGFGRKGKA**KGDIP:118 [1003 978 786 742]

17:VHRRN**T**KGDIP:27 [0 0 0 1]

YGR214W:

25:GARNVQV**∆1**HQEPYV:37 [1091 1294 1024 1193]

25:GARNVQV**V**HQEPYV:38 [0 1 0 0]

YGR214W:

25:GARNVQ**∆4**VHQEPYV:37 [1091 1294 1024 1193]

25:GARNVQ**ILFQ**VHQEPYV:41 [0 0 1 0]

YGR214W:

25:GARNVQ**VHQEPYVFNAR**PDGVHV:47 [1091 1294 1024 1193]

25:GARNVQ**∆11**PDGVHV:36 [0 1 0 0]

YHL001W:

40:IIDQKKV**L**IDGPKAGVPRQA:57 [0 0 0 0]

40:IIDQKKV**∆1**IDGPKAGVPRQA:56 [1 0 1 1]

YHR010W:

1:MAKFLKAGKV**A**VVVRGRY:18 [54 47 37 45]

1:MAKFLKAGKV**G**TVVVRGRY:19 [1 0 0 0]

YHR016C:

12:KSETK**∆5**KAAKVLR:23 [13 9 15 7]

12:KSETK**CFNYR**KAAKVLR:28 [0 1 1 3]

YIL177C:

382:SSDGIS**LLAFAGPQRNVYVDDTTR**RIQLYTD:412 [0 0 0 0]

382:SSDGIS**∆18**RIQLYTD:394 [0 0 1 0]

YIL133C:

7:VVIDG**∆12**KGHLVG:17 [342 352 322 291]

7:VVIDG**IHSFINNNLQPG**KGHLVG:29 [0 0 0 1]

YIL106W:

3:FLQN**F**HISPG:12 [13 9 10 8]

3:FLQN**∆1**HISPG:11 [0 0 0 1]

YJL225C:

382:SSDGIS**LLAFAGPQRNVYVDDTTRR**IQLYT:411 [2 7 10 10]

382:SSDGIS**∆18**RIQLYT:393 [0 0 1 0]

YJL177W:

88:VKFVQ**GLLQNAAANAE**AKGLDA:109 [330 345 410 343]

88:VKFVQ**∆11**AKGLDA:98 [0 1 0 0]

YJL136C:

4:DKGQL**VELYVPR**KCSAT:20 [578 623 614 564]

4:DKGQL∆7KCSAT:13 [0 0 0 1]

YKL180W:

98:AAANAE**∆4**AKGLDA:109 [1 0 1 2]

98:AAANAE**VCQE**AKGLDA:113 [0 1 0 0]

YKL180W:

88:VKFVQ**GLLQNAAANAE**AKGLDA:109 [1 0 1 2]

88:VKFVQ**∆11**AKGLDA:98 [0 0 0 1]

YKL157W:

88:KTSQLL**N**KTPNREIL:102 [932 994 1078 956]

88:KTSQLL**IRRTTKMTS**KTPNREIL:110 [0 3 0 0]

YKL006W:

40:DQKKV**L**IDGPKA:51 [11 8 9 10]

40:DQKKV**∆1**IDGPKA:50 [0 1 0 0]

YKL002W:

16:ERLKK**NQRALER**TQREL:32 [1933 2080 2115 1819]

16:ERLKK**∆7**TQREL:25 [0 2 0 2]

YKL002W:

16:ERLKK**NQR**ALERTQREL:32 [1933 2080 2115 1819]

16:ERLKK**∆3**ALERTQREL:29 [0 1 0 1]

YKR057W:

5:KGQL**VELYVPRKCSATNR**IIKA:26 [0 1 3 1]

5:KGQL**∆14**IIKA:12 [0 3 1 1]

YLR048W:

21:VQ**VHQEPYVFNARPDGVHVINVGKTWEKLVLAARIIAAIPNPEDVVAISSR**TY:81 [62 57 37 54]

21:VQ**∆49**TY:32 [0 1 0 2]

YLR048W:

25:GARNVQ**VHQEPYVFNARPDGVHVINVGKTWEKLVLAARIIAAIPNPEDVVAISSRTYGQRAVLKFAAHTGATPIAGRFTPGSFTNYITRSFKEPRLVIVTDPRLDAQAIKEASYVNIPVIALTDLDSPSEFVDVAIPCNNRGKHSIGLIWYLLAREVLRLRGALVDR**TQPWSIM:198 [62 57 37 54]

25:GARNVQ**∆161**TQPWSIM:37 [0 0 1 0]

YLR406C:

15:NLHKR**LHGVS**FKKRA:29 [296 329 245 285]

15:NLHKR**∆5**FKKRA:24 [0 1 1 2]

YLR426W:

17:LR**WSLSDSISICLTIYTLLINAFLIANSYIKRSGQVAWKSLREFKNGIV**LI:75 [689 792 814 774]

17:LR**F**LI:29 [0 5 7 5]

YLR464W:

166:SSDGIS**DTLTVIQSFSYSLLPVLS**ATYTSM:195 [300 346 344 366]

166:SSDGIS**∆18**ATYTSM:177 [0 0 1 0]

YLR464W:

166:SSDGIS**DTLTVIQSFSYSLLPVLSATYTSMIQQDASNCTLITTRTVHRSLD***:217 [300 346 344 366]

166:SSDGIS**RIQLYTDYNKNGSSEPRL**:189 [0 0 1 0]

YML124C:

4:VISIN**V**GQAGCQ:15 [0 0 0 0]

4:VISIN**GI**GQAGCQ:16 [0 1 0 0]

YML094W:

1:MSSQK**I**DLTKLN:12 [21 22 27 31]

1:MSSQK**LLHVRNLV**DLTKLN:19 [0 0 0 1]

YML056C:

148:SGFPVT**∆33**EDGKCP:159 [30 31 37 29

148:SGFPVT**VLLFFFFSGWAENFFSLHKVEKIKGNKYIFLNT**EDGKCP:192 [0 1 1 0]

YML034W:

633:EPEIL**WRQ**LSPTDNN:647 [10 11 13 22]

633:EPEIL**∆3**LSPTDNN:644 [0 0 0 1]

YML026C:

11:LRL**LNTNVDGNIKIVYALTTIKGVGRRYSNLVCKKADVDLHKRAGELTQEEL**ERI:69 [14 15 9 9]

11:LRL**∆49**ERI:19 [0 1 0 0]

YML026C:

11:LR**LLNTNVDGNIKIVYALTTIKGVGRRYSNLVCKKADVDLHKRAGELTQ**EELERI:39 [14 15 9 9]

11:LR**∆48**ELERI:21 [0 0 0 1]

YMR033W:

8:SIL**IIYPR**SQTTL:20 [11 24 24 16]

8:SIL**∆5**SQTTL:15 [0 0 0 1]

YMR116C:

175:DKMVK**∆1**AWNLNQ:186 [65 80 92 88]

175:DKMVK**Q**AWNLNQ:187 [0 0 1 0]

YMR201C:

1:MTPEQKAKL**EANR**KLAIERL:20 [9 11 11 10]

1:MTPEQKAKL**∆4**KLAIERL:16 [1 1 3 0]

YMR201C:

6:KAKL**EANRKLAIERLRKRGILSSDQLNRIESRNEPLKTRPLAVTSGS**NRDDN:57 [9 11 11 10]

6:KAKL**∆43**NRDDN:14 [0 0 0 1]

YMR230W:

13:QYLFQ**∆10**EGVVV:22 [39 42 50 26]

13:QYLFQ**IIELRSHIEI**EGVVV:32 [0 1 2 1]

YNL302C:

1:MAGVSV**RDVAA**QDFINAY:18 [0 0 0 0]

1:MAGVSV**S**QDFINAY:14 [1 2 0 3]

YNL302C:

4:VSV**RDVAAQD**FI:15 [0 0 0 0]

4:VSV**S**FI:9 [0 0 1 0]

YNL265C:

6:IPFT**IKLKTCLKMCIQRLRYAQEKQQAIAKQSRRQVAQLLLTNKEQKAHYRVETLIHDDIHIELLEILELYCELLLARVQVINDISTEEQLVKEHMDDGINEAIR**SLIYA:115 [241 238 274 274]

6:IPFT**S**LIYA:14 [0 1 1 0]

YNL265C:

6:IPFT**IKLKTCLKMCIQR**LRYAQ:27 [241 238 274 274]

6:IPFT**∆13**LRYAQ:14 [0 1 0 0]

YNL246W:

8:ENE**H**AKAF:15 [185 216 234 218]

8:ENE**LS**AKAF:16 [0 1 0 0]

YNL147W:

3:QQHS**K**SENK:11 [47 67 69 57]

3:QQHS**TKK**SENK:13 [0 1 0 0]

YNL038W:

194:LLFP**SNLPS**IDDQR:207 [4 3 5 7]

194:LLFP**∆5**IDDQR:202 [0 3 0 0]

YNL004W:

6:RGSE**NNNRSR**SRSRS:20 [22 46 32 32]

6:RGSE**K**SRSRS:15 [0 0 0 1]

YOL121C:

4:VSV**RDVAAQD**FINA:17 [25 32 42 20]

4:VSV**S**FINA:11 [0 0 1 0]

YOL120C:

23:NVYLK**LLVKLYTFLAR**RTDAPF:44 [420 557 488 528]

23:NVYLK**F**RTDAPF:34 [0 1 1 0]

YOR293W:

14:YLFQ**∆16**EGVVV:22 [124 89 98 94]

14:YLFQ**RDVNIILSYDNFFFYT**EGVVV:38 [0 7 4 6]

YOR293W:

14:YLFQ**E**GVVV:22 [124 89 98 94]

14:YLFQ**∆1**GVVV:21 [0 1 0 0]

YOR293W:

14:YLFQ**∆19**EGVVVAKKDFNQAKHEEIDTKNLYVIKALQSLTSKGYVKTQFSWQYYYYTLTEEGVEYLREYLNLPEHIVPGTYIQERNPTQRPQRRY*:106 [124 89 98 94]

14:YLFQ**FQRRDVNIILSYDNFFFYT**EGVVVAKKDFNQAKHEEIDTKNLYVIKALQSLTSKGYVKTQFSWQYYYYTLTEEGVEYLREYLNLPEHIVPGTYIQERNPTQRPQRRY*:125 [0 0 1 0]

YPL175W:

2:GFNI**A**MLCD:10 [89 92 105 100]

2:GFNI**VESYNNENR**MLCD:18 [0 0 1 1]

2:GFNI**ESYNNENR**MLCD:17 [0 0 0 1]

YPL031C:

3:SSSQ**∆9**FKQL:10 [40 90 26 50]

3:SSSQ**KSTSSSMNR**FKQL:19 [0 0 0 1]

YPR028W:

14:QFDT**KYSGNR**ILQQ:27 [0 0 0 0]

14:QFDT**∆6**ILQQ:21 [0 0 1 0]

YPR063C:

10:DLCVS**YKKIAPPKG**LYSAT:28 [46 29 30 26]

10:DLCVS**∆9**LYSAT:19 [0 0 0 1]

YPR063C:

10:DLCVS**YKKIAPPKGLYSATPSISGVVNQSMPMAAIFLRNKFIAWFSLIQSVHYYLNTDEDIIVAYKENKAPSPMDQPPAIKLFMSLIGLCVCYMNLV**FPQQI:111 [46 29 30 26]

10:DLCVS**∆92**FPQQI:19 [0 1 0 0]

YPR098C:

12:FYSFV**FGGTTFYSYVASPIAFKVLEKD**QFSAL:43 [164 164 160 147]

12:FYSFV**∆22**QFSAL:21 [0 1 0 0]

YPR098C:

12:FYSFV**FGGTTFYSYVA**SPIAFKVLEKDQFSAL:43 [164 164 160 147]

12:FYSFV**∆11**SPIAFKVLEKDQFSAL:32 [0 2 3 2]

YPR132W:

17:VHRRN**NRWAENNYKKRLLGTAFKSSPFGGSSHAKGIVLEKLGIESKQPNSAIRKCVRVQLIKNGKKVTAFVPNDGCLNFVDENDEVLLAGFGRKGKA**KGDIP:118 [119 130 170 114]

17:VHRRN**T**KGDIP:27 [0 0 0 1]

YPR170W-B:

41:SINDPEDGP**A**VAHTVYL:57 [11 8 27 20]

41:SINDPEDGP**VYS**VAHTVYL:59 [1 0 0 0]

YPR187W:

3:DYEE**AFNDGNENFEDFDV**EHFS:24 [1347 964 1003 789]

3:DYEE**∆14**EHFS:10 [0 0 1 0]

YPR202W:

3:IENE**QICTCIAQILHLLNSLIITFSDDDK**TETG:35 [4 16 17 11]

3:IENE**∆25**TETG:10 [0 1 0 0]
