# Supplementary material for: Widespread Use of Non-productive Alternative Splice Sites in Saccharomyces cerevisiae
Source: PLoS Genet. 2014 Apr 10;10(4):e1004249. doi: 10.1371/journal.pgen.1004249 (PMC3983031; doi:10.1371/journal.pgen.1004249)
Supplement: Figure S9 — Conservation of the intronic alternative 3′-SS in TAN1. A. Screen capture of the web browser showing the RNA-Seq reads mapped for TAN1 that use the intronic alternative 3′-SS in black, and the sequence conservation in closely related yeast species as blue peaks. B. Zoomed in view of the conservation of the sequence of the alternative intronic AAG 3′-SS. (PDF) [file pgen.1004249.s009.pdf]

A

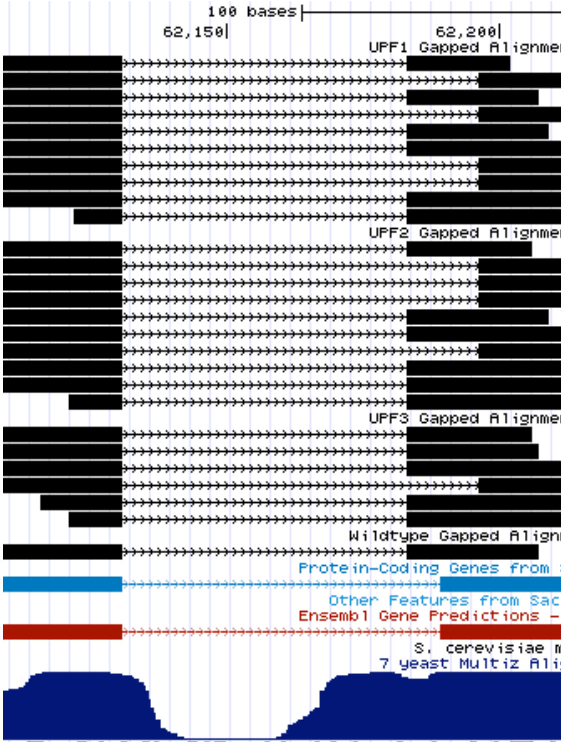

100 bases
62,150
62,200
UPF1 Gapped Alignmen
UPF2 Gapped Alignmen
UPF3 Gapped Alignmen
Wildtype Gapped Align
Protein-Coding Genes from
Other Features from Sac
Ensembl Gene Predictions –
S. cerevisiae m
7 yeast Multiz Ali

B

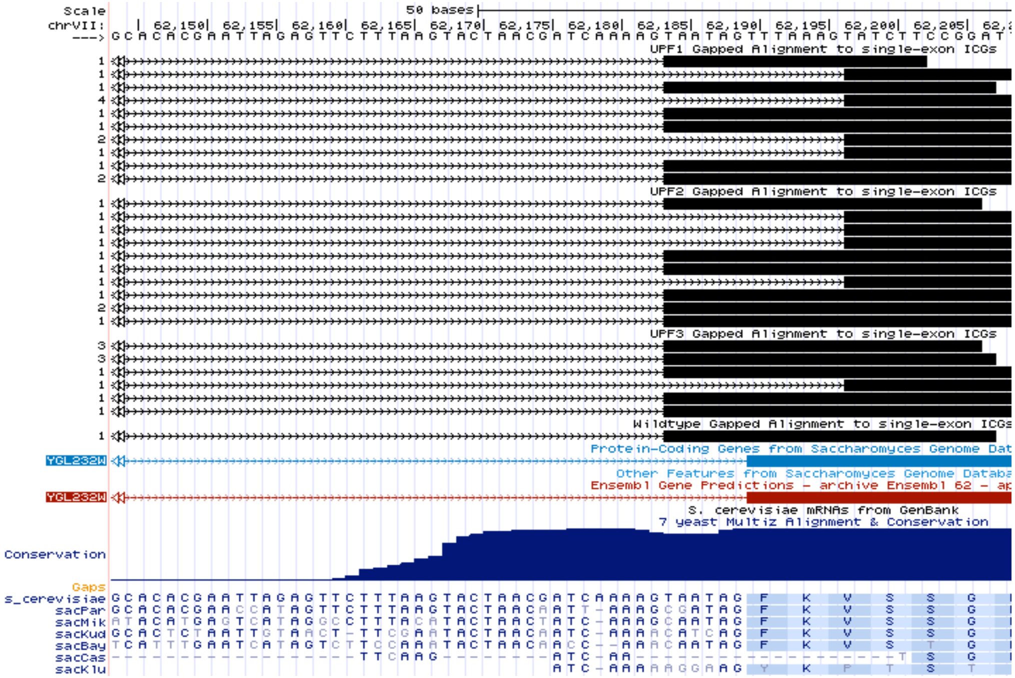

Scale
chrVII:
50 bases
UPF1 Gapped Alignment to single-exon ICGs
UPF2 Gapped Alignment to single-exon ICGs
UPF3 Gapped Alignment to single-exon ICGs
Wildtype Gapped Alignment to single-exon ICGs
YGL232W
Protein-Coding Genes from Saccharomyces Genome Dat
Other Features from Saccharomyces Genome Databa
Ensembl Gene Predictions – archive Ensembl 62 – ap
YGL232W
S. cerevisiae mRNAs from GenBank
7 yeast Multiz Alignment & Conservation
Conservation
Gaps
s_cerevisiae
sacPar
sacMik
sacKud
sacBay
sacCas
sacKlu
